# Completely non-fused electron acceptor with 3D-interpenetrated crystalline structure enables efficient and stable organic solar cell

**DOI:** 10.1038/s41467-021-25394-w

**Published:** 2021-08-24

**Authors:** Lijiao Ma, Shaoqing Zhang, Jincheng Zhu, Jingwen Wang, Junzhen Ren, Jianqi Zhang, Jianhui Hou

**Affiliations:** 1 State Key Laboratory of Polymer Physics and Chemistry, Beijing National Laboratory for Molecular, Sciences CAS Research/Education Center for Excellence in Molecular Sciences, Institute of Chemistry Chinese Academy of Sciences, Beijing, People’s Republic of China; 2 University of Chinese Academy of Sciences, Beijing, People’s Republic of China; 3 School of Chemistry and Biology Engineering, University of Science and Technology Beijing, Beijing, People’s Republic of China; 4 CAS key laboratory of nanosystem and hierarchical fabrication, CAS Center for Excellence in Nanoscience, National Center for Nanoscience and Technology, Beijing, People’s Republic of China

**Keywords:** Energy harvesting, Solar cells

## Abstract

Non-fullerene acceptors (NFAs) based on non-fused conjugated structures have more potential to realize low-cost organic photovoltaic (OPV) cells. However, their power conversion efficiencies (PCEs) are much lower than those of the fused-ring NFAs. Herein, a new bithiophene-based non-fused core (TT-P*i*) featuring good planarity as well as large steric hindrance was designed, based on which a completely non-fused NFA, A4T-16, was developed. The single-crystal result of A4T-16 reveals that a three-dimensional interpenetrating network can be formed due to the compact π–π stacking between the adjacent end-capping groups. A high PCE of 15.2% is achieved based on PBDB-TF:A4T-16, which is the highest value for the cells based on the non-fused NFAs. Notably, the device retains ~84% of its initial PCE after 1300 h under the simulated AM 1.5 G illumination (100 mW cm^−2^). Overall, this work provides insight into molecule design of the non-fused NFAs from the aspect of molecular geometry control.

## Abstract (web summary)

Non-fullerene acceptors based on non-fused conjugated structures have potential for realizing low-cost organic photovoltaic cells, owing to its synthetic simplicity. Here, the authors develop a non-fused molecule with a three-dimensional interpenetrating network and compact π-π stacking, which is highly suitable for PV applications.

## Introduction

Organic photovoltaic (OPV) cells have the outstanding advantage of fabricating large area and flexible devices through the low-cost solution coating techniques and hence attracted considerable attention in the past decades^1–3^. Benefiting from the development of various new OPV materials, especially the fused-ring non-fullerene acceptors (NFAs) with the so-called A-D-A structure, the power conversion efficiencies (PCEs) of OPV cells under the illumination of simulated solar light (AM 1.5 G, 100 mW cm^−2^) have been significantly improved^4–10^. In 2015, a new A-D-A-structured acceptor namely ITIC based on a seven fused-ring building block (DTIDT) was designed by Zhan et al., which demonstrated the possibility of utilizing NFA to realize high PCE^4,5^. Thereafter, many fused-ring building blocks were designed and applied in the molecular design of the NFAs^11–15^. In 2019, Zou et al. synthesized Y6, another new NFA featuring a more complex N-hetero-fused-ring building block, and realized even higher PCE^8^. Nowadays, although great progress in PCE has been made by employing these fused-ring-based NFAs^16–20^, the synthetic costs of the fused-ring blocks are too high to use on a relatively large scale for the future practical application^21–23^. There is still an urgent need to explore the new structural design of simple and effective molecules to meet the requirement of cost-effectiveness.

As the core units in the A-D-A-structured NFAs, the fused-ring building blocks play a critical role because they have the following characteristics. On the one hand, the conjugated backbones in these building blocks should be planar and rigid, which is beneficial for π-electron delocalization and intramolecular charge transport. As these fused-ring building blocks, all contain a few sulfur-and/or nitrogen-heteroaromatic five-membered rings and thus are electron-rich^4,8,17^, a strong intramolecular charge transfer (ICT) effect can be formed when they conjugately link with the electron-deficient end-capping groups, which endows the flexibilities in modulating absorption spectra and molecular energy levels of the NFAs^24–27^. On the other hand, the bulky alkyls on the fused-ring building blocks not only guarantee good solubilities of the NFAs^25,27,28^ but also ensure the intermolecular π−π stacking of the NFAs to mainly occur at the end-capping groups, because such a molecular packing mode is crucial for their electron-accepting capability^19,20,29–31^. Putting all of the above characteristics together, it is a big challenge to replace the fused-ring building blocks with the non-fused units.

In very recent years, great efforts have been devoted to designing the non-fused NFAs^32–38^, and the highest PCE of the OPV cells based on the completely non-fused NFAs has reached 12.76%^39^, which is still obviously lower than the state-of-the-art results for the fused-ring NFAs^40,41^. Referring to these reported works, it can be concluded that the main challenge lies in controlling the molecular geometry and packing mode of the non-fused NFAs, but the method to overcome the challenge has not been well established. From the point of view of the molecular design of non-fused acceptors, the planarity and rigidity are crucial to the aggregation and charge transfer of the acceptors. On the one hand, the more planar configuration of the molecule ensures the effective π−π stacking between the acceptors. On the other hand, the enhanced rigidity of the molecule can reduce the rotation of the C−C single bond and improve the conformational stability of the acceptors. Unlike the planar and rigid fused-rings, the non-fused units consisting of two or more small aromatic rings can be easily twisted. Therefore, in terms of molecular geometry control, the functional groups that can induce the formation of noncovalent intramolecular interactions (such as O···S, F···S, and N···S.) were commonly used to construct the planar non-fused units with higher conformation stability, which effectively enhances the photovoltaic properties of the completely non-fused NFAs such as BTIC-EH^35^, Ph-IC^32^, PTIC^33^, o-4TBC-2F^34^, etc. However, for realizing the aforementioned molecular packing mode, the functional groups with strong noncovalent intramolecular interaction cannot provide the high steric hindrance as the alkyls in the fused-ring units. Therefore, encouraged by the scientific mysteries of molecular design and the urgency of reducing the synthetic cost, it is of great necessity to develop a new method to ensure a quite planar and rigid non-fused conjugated structure and also simulate the steric hindrance of the alkyls in the fused-ring building blocks, based on which the non-fused NFAs with high photovoltaic performance will be expected.

In this contribution, we put forward a molecular design strategy by which a new bithiophene-based unit, 3,3′-bis(2,4,6-triisopropylphenyl)-2,2′-bithiophene (TT-P*i*), featuring good planarity, high conformational stability, and large steric hindrance, was obtained for forming the above-mentioned favourable molecular packing mode. Based on TT-P*i*, a new non-fused NFA (2,2′-(((3,3′′′-bis(2-ethylhexyl)-3′′,4′-bis(2,4,6-triisopropylphenyl)-[*α*-quaterthiophene]-5,5′′′-diyl)bis(methaneylylidene))bis(5,6-difluoro-3-oxo-2,3-dihydro-1H-indene-2,1-diylidene))dimalononitrile, A4T-16) was designed and synthesized, which has an optical bandgap (*E*_g_^opt^) of 1.45 eV and a lowest-unoccupied-molecular-orbital (LUMO) level of −3.96 eV. The single-crystal X-ray diffraction (XRD) results reveal that A4T-16 can form a 3D-interpenetrated network through the π−π interaction of its end-groups, which facilitates electron transport. The spin-coated film of A4T-16 exhibits a relatively high electroluminescence (EL) efficiency, implying a low non-radiative energy loss in nature. Due to these favourable properties, the A4T-16-based cells yield an outstanding PCE of 15.2%, which is not only the highest value among the OPV cells based on the non-fused electron acceptors but also at the same level as that of the fused-ring NFAs. What is more, in order to clearly demonstrate the critical role of TT-P*i* in realizing the high-performance non-fused NFA, we designed and synthesized the analogues of TT-P*i* and A4T-16 and carried out a series of comparative studies, which provide important information for the molecular design of other non-fused NFAs.

## Results and discussion

### Molecular design

**Fig. 1:**
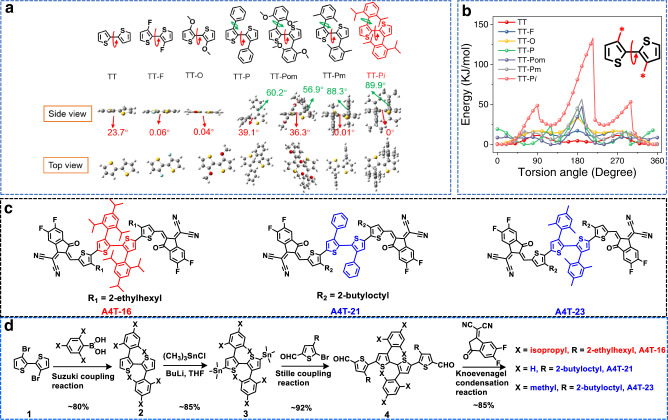
Materials design and density functional theory (DFT) calculation results. **a** Chemical structures and DFT-calculated torsion angles of the optimal geometries for several bithiophene units at the B3LYP/6-31 G (d, p) level. **b** Torsional energy profiles between two thiophenes for several bithiophene units. **c** Chemical structures of A4T-16, A4T-21, and A4T-23. **d** Synthetic route of A4T-16, A4T-21, and A4T-23.

In the established molecular design strategies, reducing the steric hindrance or enhancing the noncovalent intramolecular interaction between two non-fused aromatic rings has been widely used to obtain the planar conjugated structures. Here, we tried to realize the planar structure from the opposite direction, i.e., to obtain a planar structure with high conformational stability by introducing the functional groups with very large steric hindrance. We introduced two bulky functional groups, 2,4,6-tri-isopropylphenyl, onto the 3- and 3′-position of a bithiophene unit to get TT-P*i*, and then analyzed the optimal geometry of TT-P*i* by quantum chemistry calculation. As shown in Fig. 1a, the dihedral angel between the two thiophenes is 0^o^, implying the two thiophenes should be in one plane.

In order to prove the necessity of 2,4,6-tri-isopropylphenyl for achieving the high planarity, we calculated the optimal geometries of three representative bithiophene derivatives including bithiophene (TT), 3,3′-dimethoxy-bithiophene (TT-O) and 3,3′-difluoro-bithiophene (TT-F), and the other three analogues of TT-P*i*, including 3,3′-diphenyl-bithiophene (TT-P), 3,3′-bis(2,4,6-trimethoxylphenyl)-bithiophene (TT-Pom) and 3,3′-bis(2,4,6-trimethylphenyl)- bithiophene (TT-Pm). As shown in Fig. 1a for their optimal geometries, TT shows a dihedral angel of 23.7°, after being substituted by fluorine or methoxy, the planar conjugated structure can be formed due to the noncovalent intramolecular interactions. For TT-P and TT-Pom, the dihedral angels between the adjacent thiophenes are 39.1° and 36.3°, respectively, and these values between phenyl and thiophene are 60.2° and 56.9°, respectively. However, for TT-Pm, due to the steric hindrance caused by the methyl groups, the dihedral angel between phenyl and thiophene enlarges to 88.3°, and the torsion angle between the two thiophenes becomes smaller, i.e., 0.01°. In TT-Pm, the phenyls are nearly perpendicular to bithiophene.

In addition, we also conducted the relaxed potential surface energy scans to evaluate the conformational stabilities of the rotatable C–C bonds between the thiophenes in the seven units. As depicted in Fig. 1b, for TT, the energy difference between the stable and meta-stable states (Δ*E*_s-ms_) is about 3.0 kJ mol^−1^ and the rotation barrier from stable state to meta-stable state (*E*_s→ms_) is about 11 kJ mol^−1^. For TT-O and TT-F, due to the formation of the noncovalent intramolecular interaction, both Δ*E*_s-ms_ and *E*_s→ms_ become larger, implying these two units are more prone to maintain the optimal geometries and also have higher conformational stabilities than TT. The calculation results also reveal that to enhance the steric hindrance caused by the substituents on 2- and 4-position of the phenyl is the key to get the good planarity and high conformation stability in TT-P*i*. For TT-P*i*, Δ*E*_s-ms_ and *E*_s→ms_ reach 24 and 48 kJ mol^−1^, respectively, which are much larger than those of the other six TT derivatives, implying the planar bithiophene structure in TT-P*i* has very high conformational stability. Moreover, as shown in Fig. 1a for Van der Waals surface of TT-P*i* with the optimal geometry, the two phenyls are perpendicular to the bithiophene, and almost the whole conjugated van der Waals surface of bithiophene is shielded by the isopropyl groups due to the large steric hindrance effect of the isopropyl groups (Supplementary Fig. 2); therefore, when TT-P*i* is used as the core unit in an A-D-A-structured NFA, it can induce the formation of intermolecular π−π stacking between the end groups.

Then, we designed a non-fused NFA, namely A4T-16. The molecular structure and synthetic route are shown in Fig. 1c, d. Starting with 3,3′-dibromo-2,2′-bithiophene (compound 1), TT-P*i* is prepared by Suzuki coupling reaction with a high yield of 80% according to the previous literature^42–44^. Subsequently, a typical procedure for introducing trimethyltin groups is adopted to prepare 5,5′- bis(trimethylstannyl)-3,3′-bis(2,4,6-triisopropylphenyl)-[*α*-bithiophene] (compound 3) with a yield of 85%. Then, 3,3′′′-bis(2-ethylhexyl)-3′′,4′-bis(2,4,6-triisopropylphenyl)-[*α*-quaterthiophene]-5,5′′′-dicarbaldehyde (compound 4) can be synthesized by Stille coupling reaction between compound 3 and 5-bromo-4-(2-ethylhexyl) thiophene-2-carbaldehyde with a high yield of 92%. Finally, A4T-16 is synthesized by Knoevenagel condensation between compound 4 and 2-(5,6-difluoro-3-oxo-2,3-dihydro-1H-inden-1-ylidene)malononitrile with a yield of 85%. A4T-16 can be easily dissolved in the common solvents such as chlorobenzene, chloroform, dichloromethane, toluene, o-xylene etc. What is more, in order to prove the advantages of the chemical structure of TT-P*i*, we also designed two analogue materials, A4T-21 and A4T-23, which are synthesized by the same synthetic route as that for A4T-16. The solubilities of A4T-16, A4T-21, and A4T-23 in o-xylene are 14, 90, and 18 mg/mL, respectively. The detailed synthetic methods are provided in the “Experimental” section.

### Quantum chemistry calculation

**Fig. 2:**
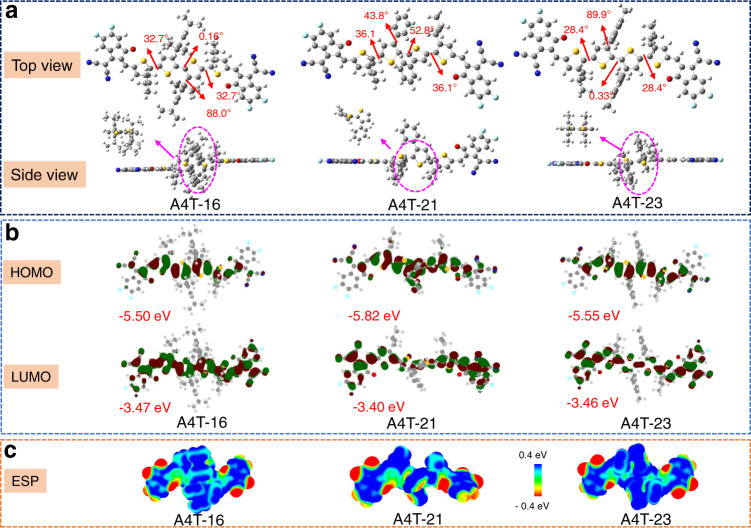
DFT calculation results of A4T-16, A4T-21, and A4T-23. **a** The optimal geometries. **b** DFT-calculated frontier molecular orbitals. **c** ESP distributions for the simplified structures of A4T-16, A4T-21, A4T-23 at the B3LYP/6-31 G(d,p) level.

To demonstrate the optimal geometry of the three NFAs, the theoretical calculations were performed via DFT calculations at the B3LYP/6-31 G (d, p) level. The ethyl-hexyl/butyl-octyl side chains were simplified to isobutyl groups. For A4T-21, the backbone is highly twisted, two end groups are in different planes, while for A4T-16 and A4T-23, although the central cores are quite planar, torsions between the core (TT-P*i*) and the alkylthiophene are observed with torsional angles of 28.4° and 30.1°, respectively. Besides, for A4T-16 and A4T-23, the phenyl groups are nearly perpendicular to the bithiophene with dihedral angles of 88.0° and 89.9°, respectively (Fig. 2a). From the calculation results, we can roughly infer that the highly twisted conjugated backbone of A4T-21 may adversely affect the molecular packing. The calculated distribution of the frontier molecular orbitals of the three acceptors is shown in Fig. 2b. The highest occupied molecular orbital (HOMO) and LUMO surface of them are well delocalized over the whole conjugated backbones, the calculated values are −5.50/−3.47, −5.82/−3.40, −5.55/−3.46 eV for A4T-16, A4T-21, and A4T-23, respectively. Moreover, their electrostatic potential (ESP) distributions are also displayed in Fig. 2c, the high ESP values suggest their excellent electron-accepting capacity. Overall, theoretical investigations manifest that A4T-16 and A4T-23 are more likely to adapt planer backbone than that of A4T-21 as the preferential conformation, which would be beneficial to ICT and intermolecular charge transport.

### Single crystal structure

**Fig. 3:**
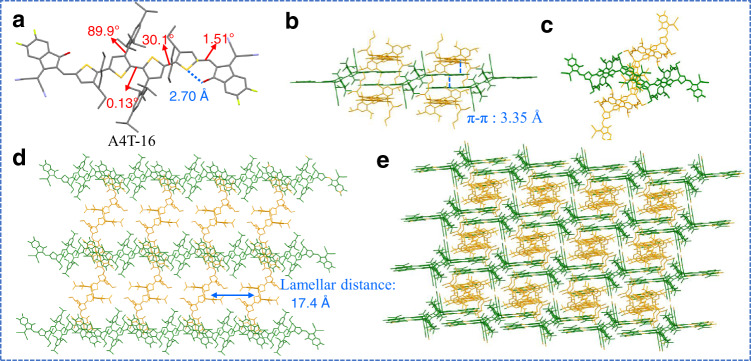
Single-crystal structure of A4T-16. **a** Single-crystal structure of A4T-16. **b** Molecules packing viewed from the *x*−*z* plane. **c** Molecules packing viewed from the *x*−*y* plane. **d** The top view of the network structure of A4T-16. **e** 3D interpenetrating network structure of A4T-16.

Single crystallographic data provide solid information for the determination of the molecular geometries and intermolecular stacking modes of organic compounds and thus have been successfully used to get insights into the correlations between aggregation structures and charge transport properties of the fused-ring-based NFAs^30,45^. After many attempts, we got the single crystal of A4T-16 via vapour diffusion method. As shown in Fig. 3a, for the single molecular configuration of A4T-16, the dihedral angle between the phenyl and thiophene is 89.9° and the dihedral angle between the two thiophenes in the core is 0.13°, which agrees well with the calculation results for TT-P*i*; the alkylthiophene is twisted relative to TT-P*i*, with a dihedral angle of 30.1°, and the dihedral angle between the alkylthiophene and the end group is 1.51°. In a single crystal, A4T-16 shows two orthogonal orientations (coloured with green and orange in Fig. 3b−e). As shown in Fig. 3b, in the in-plane horizontal direction, two adjacent A4T-16 molecules stack with each other through the end-capping groups and form a straight linear sub-structure in the crystal; by the same mode, such a sub-structure also forms in the out-plane horizontal direction. So, in these two directions, the electrons can be easily transported. The linear sub-structures in the two directions interact with each other through the end-capping groups (Fig. 3c) and form an interlocked network structure (Fig. 3d, e). In this network structure, the π−π distances between the end-capping groups are 3.35 Å, which is very compact and even smaller than those in the typical Y6-type and ITIC-type molecules^29–31^. Therefore, the crystalline structure of A4T-16 can be seen as an ideal 3D interpenetrating network for electron transport, and TT-P*i* plays a critical role in forming such a network.

### Photophysical and electrochemical properties

**Fig. 4:**
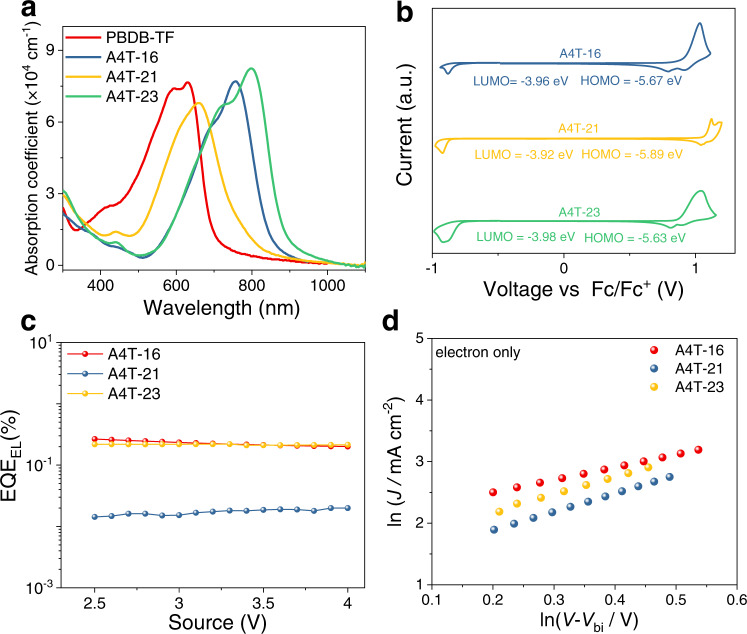
Photophysical and electrochemical properties of NFAs. **a** UV−vis absorption spectra of PBDB-TF, A4T-16, A4T-21, and A4T-23 in film states. **b** Cyclic voltammograms of A4T-16, A4T-21, and A4T-23. **c** EQE_EL_ curves of A4T-16, A4T-21 and A4T-23. **d** The electron mobility plots of the three acceptors.

The ultraviolet-visible (UV–vis) absorption spectra of the three NFAs in solid films and solution are shown in Fig. 4a and Supplementary Fig. 3, respectively. In dilute chloroform solution, the absorption spectrum of A4T-16 is almost the same as that of A4T-23, implying π-electron delocalization and molecular geometry of A4T-16 are very similar to those of A4T-23, which agree with the quantum calculation results. The molar extinction coefficients of A4T-16, A4T-21, and A4T-23 are 8.9 × 10^4^, 7.2 × 10^4^, and 1.2 × 10^5^ M^−1^ cm^−1^ at 686, 601, 686 nm, respectively (Supplementary Fig. 3c). In the solid neat films, the absorption spectra of the three materials are red-shifted. For A4T-16, the film shows an absorption peak at 756 nm and an absorption edge at about 855 nm, corresponding to an optical bandgap (*E*_g_^opt^) of 1.45 eV. *E*_g_^opt^ value of A4T-16 is slightly smaller than that of IT-4F^46^ but obviously larger than Y6^8^, these two are broadly used as fused-ring-based NFAs. The extinction coefficients of A4T-16, A4T-21, and A4T-23 films are 7.70 × 10^4^, 6.76 × 10^4^, and 8.23 × 10^4^ cm^−1^, respectively (Fig. 4a). Obviously, the absorption spectra of A4T-21 show a large hypsochromic shift than that of A4T-16 and A4T-23, which mainly resulted from the twisted backbone of A4T-21 that suppressed the ICT effect^47–49^. The detailed absorption spectral parameters of A4T-16, A4T-21, and A4T-23 are provided as Supplementary Information (SI, Supplementary Table 1). Electrochemical cyclic voltammetry (CV) measurements were performed to determine the LUMO levels of the acceptors, and as demonstrated in Fig. 4b, the LUMO levels of A4T-16, A4T-21, and A4T-23 are −3.96 −3.92, and −3.98 eV, respectively, which are slightly higher than that of IT-4F and Y6 (see Supplementary Fig. 4 for the CV curves). Overall, these three materials should be good acceptors in terms of the absorption spectra and the LUMO levels. From the point of view of molecular energy level and absorption spectrum matching, PBDB-TF, a broadly used donor material, should be a good candidate to work with the three non-fused NFAs in OPV cells.

In recent studies^50,51^, external quantum efficiencies of EL (EQE_EL_) were employed to evaluate the non-radiative energy loss (Δ*E*_non-rad_) of photovoltaic cells, because the cells with higher EQE_EL_ and thus relatively lower Δ*E*_non-rad_ have more potential in realizing higher PCEs. It has also been recognized that the EQE_EL_ of an OPV cell can be influenced by the interaction between the donor and acceptor materials, but its maximum EQE_EL_ value depends more on the component with the relatively narrower bandgap. Therefore, EQE_EL_ of the three non-fused NFAs were measured and then compared with those of the two representatives fused-ring-based NFAs, IT-4F, and Y6. As demonstrated in Fig. 4c and Supplementary Fig. 5a, A4T-16 and A4T-23 show a high EQE_EL_ of around 2.5 × 10^−3^ (Fig. 4c), and this value is very similar to that of Y6 (2.3 × 10^−3^) (Supplementary Fig. 5a). By contrast, EQE_EL_s of A4T-21 and IT-4F are 1.5 × 10^−4^ and 2.1 × 10^−4^, respectively, which are about one order of magnitude lower than that of A4T-16. The high EQE_EL_ of A4T-16 implies that it has much potential in realizing OPV cells with low *E*_loss_ values. In addition, we measured the electron mobilities (*µ*_e_) of these acceptors in parallel by space-charge-limited-current (SCLC) measurement^52^ (see Fig. 4d, Supplementary Fig. 5b and Supplementary Table 2) and found that *µ*_e_s of A4T-16 and A4T-23 are 5.5 × 10^−5^ and 2.7 × 10^−5^ cm^2^ V^−1^ S^−1^, which are quite similar to that of IT-4F (5.9 × 10^−5^ cm^2^ V^−1^ S^−1^) and Y6 (5.4 × 10^−5^ cm^2^ V^−1^ S^−1^), while *µ*_e_ of A4T-21 is about one to two times lower than them due to the twisted backbone and the poor molecular π−π stacking. Therefore, in terms of EQE_EL_ and electron mobility, A4T-16 and A4T-23 have more potential to be good NFAs.

### Photovoltaic properties, and recombination mechanism

To evaluate the photovoltaic performance of these non-fused acceptors, we fabricated the OPV cells with a device architecture of ITO/PEDOT: PSS/active layer/PF_3_N-Br/Ag. In the active layers, PBDB-TF was blended with A4T-16, A4T-21, and A4T-23, respectively. In parallel, the PBDB-TF:IT-4F- and PBDB-TF:Y6-based cells were fabricated by following the reported device fabrication procedures and used as the control devices^8,53^. The device fabrication conditions of the cells based on the three new NFAs are optimized by the commonly used processes. Here, we put the photovoltaic results of the cells fabricated via the optimal conditions together for detailed discussion.

**Fig. 5:**
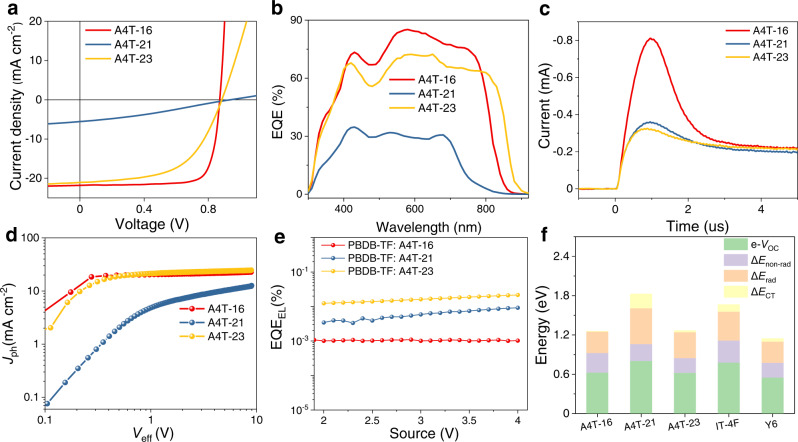
Photovoltaic properties, and recombination mechanism of the OPV cells. **a***J*−*V* curves, **b** EQE curves, **c** (Photo-CELIV) measurements for the mobilities of the fast carrier, **d** *J*_ph_−*V*_eff_ curves, **e** EQE_EL_ curves of A4T-16-, A4T-21-, and A4T-23-based cells. **f** Energy losses of A4T-16-, A4T-21-, A4T-23-, IT-4F-, Y6-based cells.

For the cells based on the three new non-fused NFAs, the current density–voltage (*J*–*V*) curves and the key parameters including PCE, open-circuit voltage (*V*_OC_), short circuit current density (*J*_SC_) and fill factor (FF) are shown in Fig. 5a and Table 1. For the control samples, the IT-4F-and Y6-based cells exhibit PCEs of 13.7% (*V*_OC_ = 0.852 V; *J*_SC_ = 21.3 mA cm^−2^; FF = 0.757) and 15.8% (*V*_OC_ = 0.841 V; *J*_SC_ = 25.5 mA cm^−2^; FF = 0.737) (Supplementary Table 3), and these photovoltaic parameters are in accordance with the reported results in literatures^8,46^. For the three non-fused NFA-based devices, the A4T-21-based cell demonstrates the highest *V*_OC_ of 0.936 V but the lowest PCE of 1.57 % due to the inferior *J*_SC_ (5.55 mA cm^−2^) and FF (0.303). The A4T-23-based cell delivers a PCE of 10.4% accompanying with a *V*_OC_ of 0.870 V, a *J*_SC_ of 21.0 mA cm^−2^, and an FF of 0.568, and such a PCE is at the same level as the state-of-the-art cells based on the completely non-fused NFAs^34,36^. Encouragingly, an impressive PCE of 15.2% (*V*_OC_ = 0.876 V, *J*_SC_ = 21.8 mA cm^−2^, FF = 0.798) is recorded for the A4T-16-based cell, which is certified as 14.8% by the National Institute of Metrology, China. (Supplementary Fig. 7). The result is not only the highest one for the non-fused NFAs but also higher than the IT-4F-based control sample. Notably, in comparison with the Y6-based control sample, the *J*_SC_ of the A4T-16-based cell is lower, because A4T-16 has a larger *E*_g_^opt^ than that of Y6, which limits the sunlight absorption capability of the A4T-16-based cell. Considering the simpler structure and the relatively high performance of these acceptors, we calculated the figure-of-merits (FoM) values of these NFAs and found that A4T-16 processes higher FoM value among these high-performance NFAs (Supplementary Table 7).

**Table 1:** Photovoltaic parameters of the A4T-16-, A4T-21-, and A4T-23-based cells.

Blends	*V*_OC_ (V)	*J*_SC_ (mA cm^−2^)	*J*_SC_ (mA cm^−2^)^a^	FF	PCE (%)^b^	*μ*_celiv_ (cm^2^ V^−1^ s^−1^)	Δ*E*_rad_ (eV)	Δ*E*_non-rad_ (eV)
PBDB-TF: A4T-16	0.876 (0.872 ± 0.003)	21.8 (21.5 ± 0.2)	21.1	0.798 (0.782 ± 0.015)	15.2 (14.8 ± 0.3)	2.4 × 10^−4^	0.326	0.298
PBDB-TF: A4T-21	0.936 (0.934 ± 0.002)	5.55 (5.32 ± 0.21)	6.91	0.303 (0.300 ± 0.003)	1.57 (1.52 ± 0.02)	1.9 × 10^−4^	0.329	0.255
PBDB-TF: A4T-23	0.870 (0.868 ± 0.002)	21.0 (20.8 ± 0.2)	20.2	0.568 (0.554 ± 0.013)	10.4 (10.1 ± 0.2)	3.0 × 10^−4^	0.363	0.227

^a^Calculated by EQE integration.

^b^Average PCE of ten devices.

The external quantum efficiency (EQE) spectra of the cells are shown in Fig. 5b and Supplementary Fig. 6b. The integrated *J*_SC_ values are 21.1, 6.91, 20.2, 20.2 and 25.3 mA cm^−2^ for the A4T-16-, A4T-21-, A4T-23-, IT-4F-, Y6-based devices, respectively, which are in accordance with those from *J*–*V* measurements. For the A4T-21-based cell, the relatively narrower response range should be ascribed to the light absorption capability of A4T-21, and the low EQE values indicate severe charge recombination in the cell. What is more, the integral current density of the A4T-21-based device is much higher than that obtained in the *J*−*V* curve; such a phenomenon indicates the hole and electron channels are not well-formed in the cell, so the severe charge recombination can be reduced to a certain degree under the weaker light intensity condition like in EQE measurement. In comparison with the A4T-16-based cell, the A4T-23-based cell shows a broader response range, which is in accord with the observations in their absorption spectra, i.e., the absorption peak of A4T-23 locates at the longer wavelength position than that of A4T-16. As the FF and EQE of the A4T-16-based cell are quite similar to the two control samples with disregarding light response range, we can conclude that charge generation and transport in the A4T-16-based cell should be efficient.

To understand the reason for the difference of the photovoltaic parameters in the three devices based on the non-fused NFAs, we analyzed their carrier transport and recombination properties. The photocurrent density (*J*_ph_) versus effective voltage (*V*_eff_) curves were used to evaluate the charge dissociation probabilities (*P*_diss_) in the cells^54^. The saturated *J*_ph_ of the A4T-16- and A4T-23-based cells are 22.9 and 24.6 mA cm^−2^ with *P*_diss_s of 95 and 85%, respectively (Fig. 5d); the *J*_ph_ values are quite high for their light absorption ranges and signify that the light-induced charge generation in the two cells are equally efficient while the charge recombination in the A4T-16-based cell is obviously lower than that in the A4T-23-based cell. For the A4T-21-based cell, the *J*_ph_ is not saturated until the *V*_eff_ reaches 10 V and the *P*_diss_ value is quite low (43.3%), which indicates inefficient charge generation and strong charge recombination. We then measured the dependence of *J*_SC_ and *V*_OC_ on light intensity (*P*_light_) to investigate the charge recombination in these cells. As plotted in Supplementary Fig. 8, the recombination parameter s in the formula *J*_SC_ ∝ *P*_light_^s^ is 0.996 for the A4T-16-based cell, implying negligible bimolecular recombination; for the A4T-23-based cell, the bimolecular recombination is slightly stronger with as value of 0.950, while for the A4T-21-based cell, a much smaller s of 0.835 indicates severe bimolecular recombination, which explains the inferior *J*_SC_ and PCE of the cell. In addition, the slopes (s′) obtained from the equation of *V*_OC_ ∝ s′*k*_B_*T*/*q*ln(*P*_light_) can be used as the indicator for estimating trap-assisted recombination^55^. As the three cells show similar slopes between 1.0 and 1.1, the trap-assisted recombination in these cells is negligible when the light intensity varies from 1 to 100 mW cm^−2^ (Supplementary Fig. 8).

To assess the charge transport properties in the three cells, we performed the photo-induced charge-carrier extraction in a linearly increasing voltage (photo-CELIV) measurements. As displayed in Fig. 5c, the mobilities of the faster carriers (*μ*_celiv_) for the A4T-16- and A4T-23-based cells are 2.4 × 10^−4^ and 3.0 × 10^−4^ cm^2^ V^−1^ S^−1^, respectively, which are at the same level as those of the highly efficient cells in previous works^56,57^, while *μ*_celiv_ of the A4T-21-based cell (1.9 × 10^−4^ cm^2^ V^−1^ S^−1^) is lower than them (Table 1). Moreover, we also fabricated the hole- and electron-only devices and measured the hole and electron mobilities (*μ*_h_ and *μ*_e_) of the three non-fused NFA-based active layers by SCLC method. As shown in Supplementary Table 2 and Supplementary Fig. 9, for the A4T-21-based active layer, a much lower *μ*_e_ (*μ*_e_ = 1.2 × 10^−5^ cm^2^ V^−1^ S^−1^) than the other two active layers is obtained and also the hole and electron transport capabilities are asymmetric, which should be the main reasons for the severe bimolecular charge recombination effect and low FF of the cell; the A4T-16-based active layer displays a highly symmetric *μ*_h_ and *μ*_e_ of 3.0 × 10^−4^ and 2.9 × 10^−4^ cm^2^ V^−1^ S^−1^, respectively, which explains the low charge recombination and thus high EQE and FF of the corresponding cell. In addition, the miscibility of the blend film was investigated, and the Flory−Huggins interaction parameters (*χ*) of PBDB-TF:A4T-16, PBDB-TF:A4T-21 and PBDB-TF:A4T-23 were calculated to be 0.52, 0.43, and 0.41, respectively (Supplementary Table 5). The smaller *χ* value indicates higher miscibility between PBDB-TF and A4T-23, which is not conducive to the formation of a purer domain. The results above can partially explain the inferior FF in PBDB-TF:A4T-23-based device^58,59^.

The Urbach energies (*E*_U_s) can be utilized to evaluate the energetic disorder of the three blends by measuring the highly sensitive EQE (s-EQE) spectra^60^. The calculated *E*_U_s are 25.4, 26.9, 26.2 meV for A4T-16-, A4T-21-, A4T-23-based devices (Supplementary Fig. 10a), smaller *E*_U_ values means a lower disorder in the blend and higher potential for achieving lower energy losses. To get insights into the *V*_OC_ difference of the three non-fused NFA-based cells, we carried out EL and high-sensitive EQE measurements to investigate the causes of energy losses. Referring to the literatures^50,51,61^, the bandgaps (*E*_g_) of the OPV cells based on the low bandgap NFAs can be determined by the cross point of the absorption and photoluminescence spectra of the NFAs. By this method, *E*_g_ of the A4T-16-, A4T-21- and A4T-23-based cells are calculated to be 1.51, 1.74, and 1.49 eV (Supplementary Fig. 10b), respectively, so the overall voltage losses which can be calculated by the equation of e*V*_loss_ = *E*_g_ − e*V*_OC_ for the three cells are 0.634, 0.804 and 0.620 eV, respectively. Based on the EQE_EL_ data shown in Fig. 5e, the Δ*E*_non-rad_ are calculated to be 0.298, 0.255 and 0.227 eV for the A4T-16-, A4T-21-, and A4T-23-based cells, respectively. As e*V*_loss_ − Δ*E*_CT_ can be separated into radiative and non-radiative energy loss (Δ*E*_rad_ and Δ*E*_non-rad_), the Δ*E*_rad_ of the A4T-16-, A4T-21-, and A4T-23-based cells are 0.326, 0.329 and 0.363 eV, respectively. The variation of Δ*E*_rad_ and Δ*E*_non-rad_ in the three blend films is a very interesting phenomenon, and the reason still needs to be revealed by combining in-depth theoretical calculation and optical physical characterization. In parallel, e*V*_loss_ − Δ*E*_CT_ /Δ*E*_rad_ /Δ*E*_non-rad_ of the IT-4F- and Y6-based cells are measured, which are 0.668/0.332/0.336 eV and 0.499/0.273/0.226 eV, respectively (Supplementary Fig. 10c and Supplementary Table 6).

Overall, both the non-radiative and radiative energy loss of the A4T-16-based cell are lower than those of the IT-4F-based cell; in comparison with the Y6-based cell, the A4T-16-based cell shows larger radiative energy loss (0.326 eV versus 0.273 eV) and non-radiative energy loss (0.298 eV versus 0.226 eV). In consideration that the non-radiative energy loss of the neat A4T-16 film is at the same level as Y6 and the non-radiative energy losses of the D: A blend films can also be tuned by changing the interaction between the donor and acceptor materials^62,63^, there should be much room for reducing the non-radiative energy loss of the A4T-16-based cells. For this purpose, PBDB-TF should be replaced by other polymer donor materials.

### Morphological characteristics

**Fig. 6:**
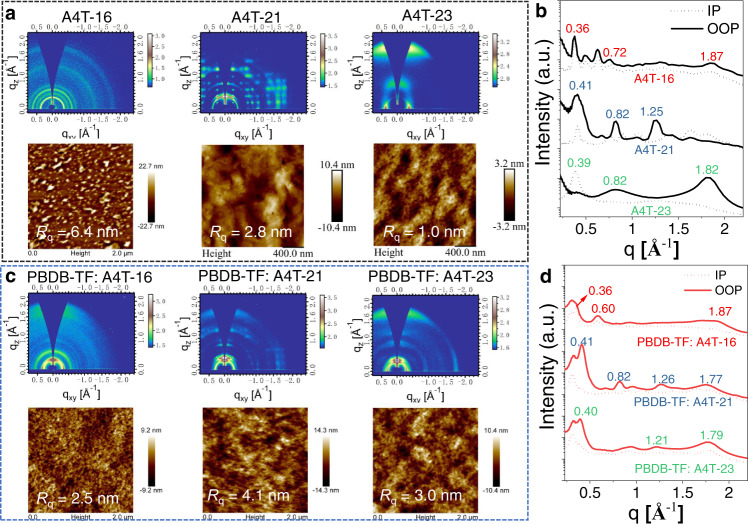
GIWAXS and AFM analysis of the blend films. **a** GIWAXS patterns and AFM height images, **b** the IP, OOP extracted line-cut profiles of the A4T-16-, A4T-21- A4T-23- pure films, **c** GIWAXS patterns and AFM height images, **d** the IP, OOP extracted line-cut profiles of the PBDB-TF: A4T-16-, PBDB-TF: A4T-21-, PBDB-TF: A4T-23- blend films.

Intermolecular packing structures of the three non-fused NFAs in the neat spin-coated films were analyzed by grazing incidence wide-angle X-ray scattering (GIWAXS). The 2D scattering patterns and the in-plane (IP) and out-of-plane (OOP) scattering profiles are illustrated in Fig. 6a, b, respectively. For the A4T-16 film, a weak (010) diffraction signal can be distinguished at 1.87 Å^−1^ in both the OOP and IP directions, corresponding to very compact π−π stacking distances of 3.35 Å; the periodical peaks at 0.36 and 0.72 are assigned to the (100) and (200) diffractions with a laminar packing distance of 17.4 Å. These values are in agreement with the single-crystal structural results (Fig. 3). Notably, the observed annular diffraction pattern is similar to that of the fullerene acceptors, suggesting that the 3D network structure mentioned above will endow more electron channels for favourable charge transfer in this acceptor. For the A4T-21 film, a typical diffraction pattern for the multi-crystal films can be observed, while (010) signal for π−π stacking is absent in OOP and IP direction. For the A4T-23 film, the face-on orientation signified by a (010) diffraction peak at 1.82 Å^−1^ at OOP direction and a (100) diffraction signal at 0.39 Å^−1^ can be found, corresponding to a π−π distance of 3.45 Å and a laminar packing distance of 16.1 Å. The above results confirm that the 3D electron transport network observed in the A4T-16 single crystal can be formed in the A4T-16 film, and also indicate the non-fused cores had a great effect on the molecular crystallinities of these acceptors.

We also analyzed the morphological properties of the blend films of PBDB-TF:A4T-16, PBDB-TF:A4T-21, and PBDB-TF:A4T-23. As shown in Fig. 6c, d for the PBDB-TF:A4T-16 blend film, the annular (100) diffraction pattern of A4T-16 still can be distinguished at the similar positions as that in the neat A4T-16 film, implying that the 3D-network of A4T-16 retained in this blend film in a certain degree. The PBDB-TF:A4T-21 films exhibited (100) lamellar peaks locating at 0.31 Å^−1^ in both the IP and OOP directions, corresponding to a *d*-spacing of 20.3 Å, and (010) diffractions only in the OOP direction (Fig. 6d), indicating a face-on-dominated molecular orientation in the film. Unlike the above two films, PBDB-TF: A4T-23 prefers to form a mixed face-on and edge-on orientations with (010) peaks in OOP and IP directions. The (010) peaks of the PBDB-TF:A4T-16, PBDB-TF:A4T-21, and PBDB-TF:A4T-23 films are at 1.87, 1.77, and 1.79 Å^−1^, corresponding to the π−π distances of 3.36, 3.55, and 3.51 Å, respectively. Obviously, the more compact π−π distance can be formed in the PBDB-TF:A4T-16 film than the other two blends. The π−π distance in PBDB-TF:A4T-23 blend film is larger than that of in A4T-23 neat film, implying the molecular orientation of A4T-23 is more significantly affected by PBDB-TF compared with A4T-16. The face-on orientation and π−π packing distance of A4T-23 are not well retained in the A4T-23-based blend film. In addition, the higher miscibility between PBDB-TF and A4T-23 aforementioned may also be responsible for the decreased FF in the OPV cell.

As depicted in Fig. 6a and Supplementary Fig. 12, by employing chloroform as the processing solvent for spin-coating, the neat A4T-16 film demonstrates high surface roughness (*R*_q_) of 6.4 nm with large aggregations, while the A4T-21 and A4T-23 films show much smoother surfaces with the *R*_q_ of 2.8 and 0.5 nm, respectively. The PBDB-TF:A4T-16-, PBDB-TF:A4T-21-, and PBDB-TF:A4T-23-based films demonstrate relatively smooth surfaces with the *R*_q_ of 2.5, 4.1, and 3.0 nm (Fig. 6c), and the nanoscale phase separation can be formed in these blends (Supplementary Fig. 12b). Therefore, generally speaking, the three non-fused NFAs have no problem in realizing the favourable phased separation when blended with PBDB-TF. In transmission electron microscope (TEM) measurement, there was no significant difference in phase separation of the three non-fused blend films (Supplementary Fig. 13).

### Universality and device stability of A4T-16

**Fig. 7:**
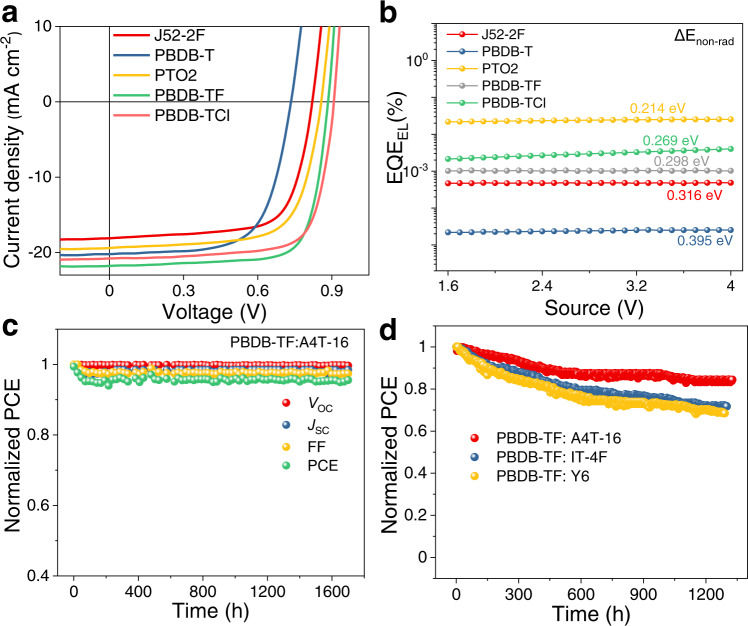
Universality of A4T-16 and device stability. **a***J*−*V* curves, **b** EQE_EL_ curves of polymer donors: A4T-16-based cells. **c** The storage stability of the encapsulated PBDB-TF: A4T-16 cell (the encapsulated cells were stored in a glovebox under the nitrogen atmosphere). **d** Photo-stability of IT-4F-, Y6- and A4T-16-based cells (the encapsulated cells were measured in air under the illumination of AM 1.5 G, 100 mW cm^−2^ at ~50 ± 3 °C).

Here, we selected a few representative polymer donors including PBDB-T, J52-2F, PTO2 and PBDB-TCl (Supplementary Fig. 14) to blend with A4T-16 and then fabricated a series of OPV cells. The aforementioned device architecture and the same fabrication conditions for the PBDB-TF:A4T-16-based cell are adopted without any further optimization. The *J*−*V* and EQE_EL_ curves and the photovoltaic parameters are shown in Fig. 7a, b and Table 2.

**Table 2:** Photovoltaic parameters of the polymers: A4T-16-based cells.

Donors	*V*_OC_ [V]	*J*_SC_ [mA cm^−2^]^a^	FF	PCE^b^ (%) [max (average)]	Δ*E*_non-rad_ (eV)	EQE_EL_
J52-2F	0.821	18.1 (17.9)	0.699	10.4 (10.1 ± 0.2)	0.316	4.86 × 10^−6^
PBDB-T	0.735	20.9 (20.3)	0.643	9.88 (9.50 ± 0.31)	0.395	2.35 × 10^−7^
PBDB-TF	0.876	21.8 (21.1)	0.798	15.2 (14.8 ± 0.3)	0.298	1.04 × 10^−5^
PBDB-TCl	0.909	20.8 (20.5)	0.740	14.0 (13.7 ± 0.2)	0.269	2.99 × 10^−5^
PTO2	0.952	18.7 (17.9)	0.665	11.8 (11.0 ± 0.5)	0.214	2.50 × 10^−4^

^a^Calculated by EQE integration.

^b^Average PCE of ten devices.

It should be also noted that, the device fabrication method that inherits from the PBDB-TF:A4T-16 cells may not be optimal for making the cells with the other polymers, so the photovoltaic results in this section can only be used to make a preliminary evaluation for the universality of A4T-16. Nevertheless, it is very clear that no matter which donor is used, the cells exhibit decent *J*_SC_s and FFs. In addition, for these cells, although the EQE onsets at the long-wavelength direction are the same (Supplementary Fig. 15a), the *V*_OC_ values vary from 0.735 to 0.952 V, meaning the *V*_loss_ in these devices may be quite different. Therefore, we measured the EL properties and then calculated the Δ*E*_non-rad_ values of the cells. As shown in Fig. 7b and Table 2, for the PTO2:A4T-16-based cell, the Δ*E*_non-rad_ value is as low as 0.214 eV, which is even smaller than the aforementioned Y6-based cell; for the PBDB-T:A4T-16-based cell, this value is as large as 0.395 eV, resulting in a low *V*_OC_ of 0.735 V. Overall, these results clearly demonstrate that A4T-16 is an excellent non-fused acceptor with good universality and has potential to achieve higher photovoltaic performance.

In addition to high PCE, the long-term device stabilities of OPV cells in storage and under the continuous sunlight illumination are very important to their practical application in the future. Here, we studied the storage stability of the encapsulated PBDB-TF:A4T-16 cell and found that the cell maintains 95% of its initial PCE after 1700 h storage under the nitrogen atmosphere with a glovebox temperature of 22 ± 5 °C. (Fig. 7c). Furthermore, the device stability of the cell under the continuous 1-sun-equivalent illumination was investigated. In order to make clear comparisons, we also tested the stabilities of the PBDB-TF:IT-4F- and PBDB-TF:Y6-based cells in parallel. It is worth noting that this part of stability measurements was carried out under the ambient atmosphere with 40 ± 10% humidity, and the encapsulated cells were naturally heated up to 50 ± 3 °C by the continuous illumination. As shown in Fig. 7d, the PBDB-TF:A4T-16-based cell retains ~84% of its initial PCE after 1300 h illumination, which is higher than those of the two control samples, i.e., 72% for the PBDB-TF:IT-4F-based cell and 71% for the PBDB-TF:Y6-based cell.

To explore the cause for the different stabilities of the three cells, the intrinsic photochemical stabilities of A4T-16, IT-4F, and Y6 were evaluated by exposing the neat films under the continuous illumination by a 365 nm UV-light and a solar simulator under the ambient atmosphere. The UV−vis absorption spectra of the neat films were monitored and used as the indicator for their photochemical stabilities. As shown in Supplementary Fig. 16, under 365 nm UV-light, for the absorption spectra of A4T-16, A4T-23, and Y6 films, the absorbances slightly decreased after 8 days. However, for the IT-4F and A4T-21 films, the absorption spectrum greatly changed after 8 days of illumination and are completely bleached after ~16 days. Under continuous 1-sun-equivalent illumination, the absorbance of the IT-4F and A4T-21 films decreased rapidly in the first 3 h, and no absorption can be detected after 17 h, implying the degradation of IT-4F and A4T-21. By contrast, the absorbance of Y6, A4T-23, and A4T-16 decreases slowly, with a low attenuation after 25 h (Supplementary Fig. 17). Although further studies on the photochemical stability of these acceptors are still needed, the current results already demonstrate the superiority of A4T-16 and Y6 in realizing stable and efficient OPV cells.

In summary, we designed a bithiophene-based building block (TT-P*i*) with a unique molecular geometry, based on which a new non-fused NFA with the superior properties for photovoltaic application was prepared. In TT-P*i*, 2,4,6-trimethylphenyl, a functional group with very high and symmetric steric hindrance, was used to lock the two thiophenes into one plane. The DFT calculation results reveal that this new method is not only more effective in enhancing the conformational stability of the planar structure than the broadly used noncovalent conformational lockers like O···S and S···F, but also provide the steric hindrance for inducing the formation of the favourable intermolecular packing mode of the A-D-A-structured NFAs. The single-crystal structure of A4T-16 shows the end-capping groups of the adjacent molecules stack in the perpendicular and horizontal direction with a very compact π−π distance of 3.35 Å, by which a 3D interpenetrated network can be formed. GIWAXS measurements demonstrate that the 3D network of A4T-16 could be partially retained in the blend film.

The PBDB-TF:A4T-16-based cell not only yields a PCE of 15.2%, the highest value for the cells based on non-fused NFAs, but also exhibits excellent long-term stability under the continuous simulated 1-sun-illumination due to the superior photochemical stability of the A4T-16. Device physics studies indicate the PBDB-TF:A4T-16-based OPV cells have less energetic disorder than the analogues, and by blending with the other polymer donors, the non-radiative energy loss of the A4T-16-based cells can be as low as 0.214 eV, which is even smaller than that of the Y6-based cell. The photovoltaic characterizations also reveal that A4T-16 has good universality. Overall, the molecular design method and the superior photovoltaic properties of A4T-16 can be used as the guidance for developing new non-fused NFAs with the merits of high efficiency, high stability, and low cost.

### Reporting summary

Further information on research design is available in the Nature Research Reporting Summary linked to this article.

## Supplementary information

Supplementary Information

Solar Cells Reporting Summary

## Data Availability

The data that support the findings of this study are available from the corresponding author on request. The X-ray crystallographic coordinate for A4T-16 reported in this study has been deposited at the Cambridge Crystallographic Data Centre (CCDC), under deposition numbers 2074697. These data can be obtained free of charge from The Cambridge Crystallographic Data Centre via www.ccdc.cam.ac.uk/data_request/cif.
