# Supplementary material for: Completely non-fused electron acceptor with 3D-interpenetrated crystalline structure enables efficient and stable organic solar cell
Source: Nat Commun. 2021 Aug 24;12:5093. doi: 10.1038/s41467-021-25394-w (PMC8384863; doi:10.1038/s41467-021-25394-w)
Supplement: Supplementary file 1 — Supplementary Information [file 41467_2021_25394_MOESM1_ESM.pdf]

# SUPPLEMENTARY INFORMATION

**Completely Non-Fused Electron Acceptor with 3D-interpenetrated Crystalline Structure Enables Efficient and Stable Organic Solar Cell**

*Lijiao Ma et al.*

*State Key Laboratory of Polymer Physics and Chemistry, Beijing National Laboratory for Molecular, Sciences CAS Research/Education Center for Excellence in Molecular Sciences, Institute of Chemistry Chinese Academy of Sciences, Beijing 100190, P. R. China*

## SUPPLEMENTARY METHODS

### *Materials.*

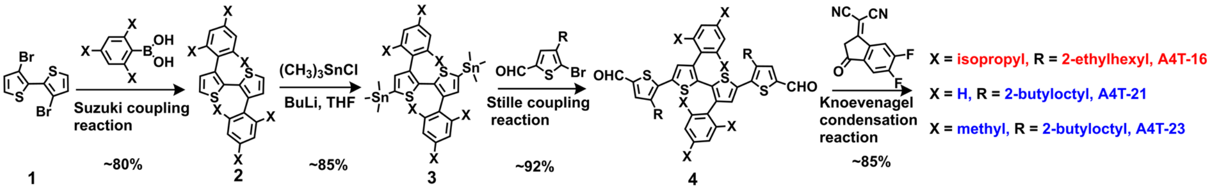


Compound 1, PBDB-TF, IT-4F, Y6, PBDB-TCl, PTO-2, J52-2F, PBDB-T, PFN-Br, 5,6-difluoro-3-(dicyanomethylidene)indan-1-one, 5-bromo-4-(2-ethylhexyl)thiophene-2-carbaldehyde, and 5-bromo-4-(2-butyloctyl)thiophene-2-carbaldehyde were purchased from Solarmer Materials Inc. The number-average molecular weight ($M_n$) of polymer PBDB-TF was about 35 kDa, with a polydispersity index (PDI) of 2.77. Phenylboronic acid, 2,4,6-trimethylbenzeneboronic acid, and 2,4,6-triisopropylphenylboronic acid were purchased from Innochem. PEDOT: PSS, Clevios™ P VP AI 4083, was purchased from Heraeus. The other reagents and solvents were commercially available and used as received.

A4T-16, A4T-21 and A4T-23 were obtained by the same synthetic process as follows, and here we provide the synthesis procedure of A4T-16 as the example.

***3,3'-bis(2,4,6-triisopropylphenyl)-2,2'-bithiophene (compound 2)***

Compound 2 was obtained by Suzuki coupling according to the previous literature [1-3], and the detailed synthesis procedure is as follows. Compound 1 (1.46 g, 4.5 mmol), 2,4,6-triisopropylphenylboronic acid (4.47 g, 18 mmol) and $Pd(PPh_3)_4$ (312 mg, 0.27 mmol) was dissolved in 55 mL of DME in a 250 mL flask under inert atmosphere. Then, a solution of KO$^t$Bu (3.53 g, 31.5 mmol) in $^t$BuOH (22 mL) was added into the flask via syringe. The mixture was stirred and refluxed for 96 hours under nitrogen protection. Please note that the reaction for 3,3'-diphenyl-2,2'-bithiophene and 3,3'-dimesityl-2,2'-bithiophene (the intermediates of A4T-21 and A4T-23, respectively) is about 1 hour, which is much shorter than that of compound 2. After cooling the reactant to room temperature, a mixed solution of $H_2O$ (10 mL) and methanol (50 mL) was added, and the raw product can be precipitated and filtered. The product was then purified by silica gel column chromatography with petroleum ether (PE):dichloromethane (DCM)=2:1 as eluent and obtained as light yellow solid with a yield of 84%.

$^1$H NMR (300 MHz, $CDCl_3$) (ppm): δ 7.10 (s, 4H), 6.99 (d, J = 5.1 Hz, 2H), 6.70 (d, J = 5.1 Hz, 2H), 2.99 (dt, J = 13.8, 6.9 Hz, 2H), 2.63 (dt, J = 13.7, 6.8 Hz, 4H), 1.35 (d, J = 6.9 Hz, 12H), 1.05 (dd, J =

11.2, 6.9 Hz, 24H). $^{13}$C NMR (75 MHz, $CDCl_3$) (ppm): δ 149.29, 147.86, 136.61, 132.57, 131.55, 129.86, 123.34, 121.28, 34.36, 30.53, 25.20, 24.26, 23.25.

***(3,3'-bis(2,4,6-triisopropylphenyl)-[2,2'-bithiophene]-5,5'-diyl)bis(trimethylstannane) (compound 3)***

The solution of compound 2 (1.56 g, 2.73 mmol) in dried tetrahydrofuran (THF, 30 mL) was cooled to -40 °C, and n-BuLi (2.5 M, 6.29 mmol) was added dropwise. The mixture was stirred at -40 °C for 1 h and then $Me_3SnCl$ (1.0 M, 6.83 mmol) was added and stirred for another 1 h. The mixture was then poured into water and extracted with DCM. The product was purified by recrystallization from isopropanol and obtained as light-yellow solid with a yield of 85%.

$^1$H NMR (300 MHz, $CDCl_3$) (ppm): δ 7.07 (s, 4H), 6.75 (s, 2H), 3.14 – 2.81 (m, 2H), 2.57 (dt, *J* = 13.6, 6.8 Hz, 4H), 1.33 (d, *J* = 6.9 Hz, 12H), 1.02 (dd, *J* = 6.8, 3.2 Hz, 24H), 0.18 (s, 18H). $^{13}$C NMR (75 MHz, $CDCl_3$) (ppm): δ 149.09, 147.88, 138.52, 137.97, 137.52, 134.62, 132.17, 121.07, 34.59, 30.39, 25.15, 24.38, 23.14.

***3,3'''-bis(2-ethylhexyl)-3'',4'-bis(2,4,6-triisopropylphenyl)-[2,2':5',2'':5'',2'''-quaterthiophene]-5,5''' dicarbaldehyde (compound 4)***

Compound 4 was obtained by Stille coupling of 5-bromo-4-(2-ethylhexyl)thiophene-2-carbaldehyde (1.5 mmol, 454 mg) and compound 3 (0.5 mmol, 448 mg), with Pd $(PPh_3)_4$ (60 mg) as the catalyst. The raw product was purified with chromatography column by using PE/DCM (5:1) as eluent, and compound 4 was obtained as orange solid with a yield of ~90%.

$^1$H NMR (300 MHz, $CDCl_3$) (ppm): δ 9.77 (s, 2H), 7.50 (s, 2H), 7.15 (s, 4H), 6.94 (s, 2H), 3.00 (m, *J* = 13.8, 6.9 Hz, 2H), 2.84 – 2.59 (m, 4H), 2.50 (t, *J* = 16.6 Hz, 4H), 1.57 (d, *J* = 4.3 Hz, 2H), 1.36 (d, *J* = 6.9 Hz, 12H), 1.16 (m, *J* = 21.2, 14.6, 7.5 Hz, 40H), 0.94 – 0.60 (m, 12H).

$^{13}$C NMR (75 MHz, $CDCl_3$) (ppm): 182.51, 150.32, 147.67, 141.71, 140.50, 139.60, 138.98, 138.42, 133.96, 133.36, 130.25, 121.70, 40.36, 34.44, 33.22, 32.37, 30.76, 28.70, 25.65, 25.19, 24.23, 23.34, 22.94, 14.03, 10.71.

MS (MALDI-TOF): m/z 1013.6

***2,2'-((2Z,2'Z)-((3,3'''-bis(2-ethylhexyl)-3'',4'-bis(2,4,6-triisopropylphenyl)-[2,2':5',2'':5'',2'''-quaterthiophene]-5,5'''-diyl)bis(methanylylidene))bis(5,6-difluoro-3-oxo-2,3-dihydro-1H-indene-2,1-diylidene))dimalononitrile (A4T-16)***

Compound 4 (121.8 mg, 0.12 mmol) and 5,6-difluoro-3-(dicyanomethylidene)indan-1-one (111.5 mg, 0.48 mmol) were dissolved in chloroform (6 mL), and pyridine (0.1 mL) were added into the mixture. After the reaction finished, the solvent was removed under a reduced pressure and the residue was purified by chromatography on silica gel with PE/DCM (1:1) to give A4T-16 (137.8 mg) as a black solid with a yield of 80%.

$^1$H NMR (300 MHz, $CDCl_3$) (ppm): δ 8.69 (s, 2H), 8.54 (dd, *J* = 10.0, 6.5 Hz, 2H), 7.64 (dd, *J* = 14.5, 7.0 Hz, 4H), 7.22 – 7.19 (d, 6H), 3.04 (dt, *J* = 13.7, 6.8 Hz, 2H), 2.70 (dt, J = 13.3, 6.6 Hz, 4H), 2.53 (d, J = 7.2 Hz, 4H), 1.61 (s, 2H), 1.38 (d, J = 6.9 Hz, 12H), 1.32 – 1.02 (m, 40H), 0.93 – 0.70 (m, 12H).

$^{13}$C NMR (75 MHz, $CDCl_3$) (ppm): δ 185.57, 158.38, 150.51, 149.90, 148.45, 147.56, 140.74, 139.65, 137.43, 136.10, 135.66, 135.51, 134.56, 133.92, 131.72, 129.74, 121.74, 115.10, 114.81, 114.17, 112.50, 69.86, 40.27, 34.42, 32.80, 30.90, 28.73, 25.78, 25.21, 24.19, 23.48, 22.99, 14.06, 10.81.

MS (MALDI-TOF): m/z 1438.8.

***Synthesis of A4T-21***

The synthesis procedure is the same as A4T-16 by replacing 2,4,6-triisopropylphenyl with phenyl.

***3,3'-diphenyl-2,2'-bithiophene*** (yield 80%)

$^1$H NMR (300 MHz, $CDCl_3$) (ppm): δ 7.34 (d, J = 5.2 Hz, 2H), 7.16 – 7.10 (m, 6H), 7.08 (d, J = 5.2 Hz, 2H), 7.06 – 7.00 (m, 4H). $^{13}$C NMR (75 MHz, $CDCl_3$) (ppm): δ 141.20, 135.96, 130.06, 129.36, 128.39, 128.04, 126.61, 125.99.

***(3,3'-diphenyl-[2,2'-bithiophene]-5,5'-diyl) bis (trimethylstannane)*** (yield 85%)

$^1$H NMR (300 MHz, $CDCl_3$) (ppm): δ 7.14 – 7.04 (m, 8H), 7.00 (dd, J = 6.5, 3.1 Hz, 4H), 0.38 (s, 18H). $^{13}$C NMR (75 MHz, $CDCl_3$) (ppm): δ 141.53, 138.48, 137.51, 136.39, 136.33, 128.32, 127.92, 126.17.

***3,3'''-bis(2-butyloctyl)-3'',4'-diphenyl-[2,2':5',2'':5'',2'''-quaterthiophene]-5,5'''-dicarbaldehyde*** (yield 82%)

$^1$H NMR (400 MHz, $CDCl_3$) (ppm) δ 9.84 (s, 2H), 7.55 (s, 2H), 7.26 (d, *J* = 1.9 Hz, 2H), 7.24 – 7.15 (m, 6H), 7.15 – 7.07 (m, 4H), 2.73 (d, *J* = 7.3 Hz, 4H), 1.71 (s, 2H), 1.25 (d, *J* = 3.7 Hz, 32H), 0.88 – 0.85 (m, 12H).

$^{13}$C NMR (125 MHz, $CDCl_3$) (ppm) δ 182.57, 142.09, 140.80, 140.58, 140.17, 139.47, 135.51, 135.01, 130.97, 129.78, 128.37, 127.33, 38.76, 34.00, 33.38, 33.08, 31.84, 29.68, 28.68, 26.44, 23.04, 22.66, 14.11.

MS (MALDI-TOF): m/z 874.4

***2,2'-((2E,2'E)-((3,3'''-bis(2-butyloctyl)-3'',4'-diphenyl-[2,2':5',2'':5'',2'''-quaterthiophene]-5,5'''-diyl)bis(methanylylidene))bis(5,6-difluoro-3-oxo-2,3-dihydro-1H-indene-2,1-diylidene))dimalononitrile (A4T-21)*** (yield 84%)

$^1$H NMR (400 MHz, $CDCl_3$) (ppm) δ 8.79 (s, 2H), 8.55 (dd, 2H), 7.68 (dd, 4H), 7.47 (s, 2H), 7.21 (qd, 6H), 7.13 – 7.07 (m, 4H), 2.76 (d, 4H), 1.74 (s, 2H), 1.25 (d, 32H), 0.86 (t, 12H).

$^{13}$C NMR (125 MHz, $CDCl_3$) (ppm) δ 185.97, 158.21, 155.83, 153.28, 149.09, 148.84, 142.78, 140.90, 137.75, 136.60, 135.54, 134.71, 134.45, 132.55, 130.95, 128.45, 127.58, 122.04, 114.03, 112.75, 38.54, 33.89, 33.41, 33.09, 31.86, 29.71, 28.64, 26.43, 23.07, 22.68, 14.13.

MS (MALDI-TOF): m/z 1298.4

***Synthesis of A4T-23***

The synthesis procedure is the same as A4T-16 by replacing 2,4,6-triisopropylphenyl with 2,4,6-trimethylphenyl.

***3,3'-dimesityl-2,2'-bithiophene*** (yield 85%)

$^1$H NMR (300 MHz, $CDCl_3$) (ppm): δ 7.07 (d, J = 5.1 Hz, 2H), 6.97 (s, 4H), 6.91 – 6.81 (m, 2H), 2.38 (s, 6H), 1.97 (s, 12H). $^{13}$C NMR (75 MHz, $CDCl_3$) (ppm): δ 137.87, 137.81, 137.19, 133.55, 131.97, 128.90, 128.50, 124.44, 21.30, 20.27.

***(3,3'-dimesityl-[2,2'-bithiophene]-5,5'-diyl)bis(trimethylstannane)***(yield 87%)

$^1$H NMR (300 MHz, $CDCl_3$) (ppm): δ 6.87 (s, 4H), 6.69 (s, 2H), 2.34 (s, 6H), 1.85 (s, 12H), 0.26 (s, 18H). $^{13}$C NMR (75 MHz, $CDCl_3$) (ppm): δ 138.69, 137.79, 137.70, 137.28, 137.01, 136.53, 133.78, 128.15, 77.35, 77.03, 76.71, 21.12, 20.38.

***3,3'''-bis(2-butyloctyl)-3'',4'-dimesityl-[2,2':5',2'':5'',2'''-quaterthiophene]-5,5'''-dicarbaldehyde*** (yield 85%)

$^1$H NMR (400 MHz, $CDCl_3$) (ppm) δ 9.79 (s, 2H), 7.50 (s, 2H), 7.02 (s, 4H), 6.94 (s, 2H), 2.56 (d, J = 7.2 Hz, 4H), 2.40 (s, 6H), 2.07 (s, 12H), 1.61 (s, 2H), 1.40 – 1.08 (m, 32H), 1.00 – 0.76 (m, 12H).

$^{13}$C NMR 125 MHz, $CDCl_3$) (ppm) δ 181.44, 140.60, 139.14, 138.62, 138.32, 137.46, 136.53, 133.36, 132.34, 131.22, 128.48, 127.91, 38.01, 32.92, 32.33, 31.99, 30.83, 28.64, 27.67, 25.49, 22.01, 21.64, 20.32, 19.29, 13.08.

MS (MALDI-TOF): m/z 958.5

***2,2'-((2E,2'E)-((3,3'''-bis(2-butyloctyl)-3'',4'-dimesityl-[2,2':5',2'':5'',2'''-quaterthiophene]-5,5'''-diyl)bis(methanylylidene))bis(5,6-difluoro-3-oxo-2,3-dihydro-1H-indene-2,1-diylidene))dimalononitrile (A4T-23)*** (yield 85%)

$^{1}$H NMR (400 MHz, $CDCl_3$) (ppm) δ 8.73 (s, 2H), 8.53 (dd, *J* = 10.0, 6.5 Hz, 2H), 7.63 (dd, *J* = 21.9, 14.4 Hz, 4H), 7.23 (s, 2H), 7.05 (s, 4H), 2.54 (d, *J* = 7.3 Hz, 4H), 2.41 (d, *J* = 11.8 Hz, 6H), 2.08 (s, 12H), 1.63 (s, 2H), 1.25 (dd, *J* = 23.7, 6.7 Hz, 32H), 0.88 (t, *J* = 6.9 Hz, 12H).

$^{13}$C NMR 125 MHz, $CDCl_3$) (ppm) δ 185.86, 158.33, 155.71, 153.26, 153.1, 149.92, 148.92, 140.77, 139.74, 138.82, 137.46, 135.16, 134.92, 134.62, 131.32, 131.79, 131.00, 129.10, 121.57, 115.1, 114.9, 114.13, 112.69, 112.51, 69.91, 39.11, 33.81, 33.39, 33.04, 31.87, 29.73, 28.74, 26.56, 23.08, 22.67, 21.38, 20.33, 14.12.

MS (MALDI-TOF): m/z 1382.5

***Materials characterization***

The molecular energy levels were measured by utilizing CHI650D Electrochemical Workstation via square wave voltammetry methods. The working electrode, counter electrode, and reference electrode were Glassy carbon disk, Pt wire, and an $Ag/Ag^{+}$ electrode respectively with the ferrocene/ferrocenium ($Fc/Fc^{+}$) as an external standard in the measurement. HOMO/LUMO = –e (φox/φred + 4.80–φ$Fc/Fc^{+}$) (eV). Absorption spectra were measured on a Hitachi UH4150 UV-vis spectrophotometer. $^{1}$HNMR, $^{13}$CNMR spectra were recorded on BRUKER Fourier 300 and 400 spectrometer.

***Solubility measurement***

To 100 mg of non-fused acceptors, 1 mL of o-xylene was added. The solution was stirred for 3 h at room temperature. The solution was filtered by centrifugation. To 0.5 mL of the filtrate, methanol was added until all solids precipitated out. The precipitate was extracted and dried under vacuum overnight. The solubility of the acceptors can be calculated by the following equation.

solubility (mg/mL) = weight of the precipitate (mg) /0.5 mL

***Single-Crystal growth.***

A solution prepared from ~1 mg A4T-16 in ~0.5 mL toluene was transferred into a 4 mL vial, which was capped with a bottle cap. The 4 mL vial was then placed in a 20 mL vial containing ~3 mL petroleum ether. The 20 mL vial was then tightly sealed, and left standing for a few days (5-7 days) to give rod-shaped crystal clusters.

***Device fabrication***

A conventional structure of ITO/PEDOT:PSS/active layer/$PF_3N$-Br/Ag was used to fabricate the OSCs. ITO-coated glass was purchased from South China Xiang's Science & Technical Company Limited and washed ultrasonically in water/detergent, water, acetone and isopropanol in sequence for 15 min. After 15 min of ultraviolet-ozone treatment, the PEDOT:PSS was spin-coated on the ITO glasses at 3,000 r.p.m., then the ITO substrates were treated by thermal annealing for 15 min at 150 °C. The active layer was spin-coated on the PEDOT: PSS layer via spin-coating, The concentration of the PBDB-TF, PBDB-TCl: A4T-16 (1:1.2) blend solution for spin-coating was 10 mg/mL (polymer/ o-xylene), 1,8-diiodooctane (DIO) as additive with 0.5% volume ratio was added prior to spin-coating process. PBDB-T:A4T-16 (1:1.2), J52-2F: A4T-16 (1:1.2), PTO-2:A4T-16 (1:1.2) were dissolved in chlorobenzene at a polymer concentration of 10 mg $mL^{-1}$. To dissolve the polymers fully, the active-layer solution was stirred at 45 °C for 2 h, DIO was added to the blend solution 30 min before the spin-coating process with a ratio of 0.5% (v:v) to the host solvent CB. The resultant film thickness was obtained via a surface profilometer (Dektak XT, Bruker). After spin-coating the active layer solutions, A thin PFN-Br layer was spin-coated on the active layer at 3,000 r.p.m. from the alcohol solution. Finally, about 100-nm-thick Ag was evaporated onto the active layer under high vacuum (~$9\times10^{-4}$ Pa).

***Device characterization and measurement***

The *J*−*V* measurement was performed via a XES-70S1 (SAN-EI Electric Co., Ltd.) solar simulator (AAA grade) whose intensity was calibrated by a certified standard silicon solar cell (SRC-2020, Enlitech) under illumination of AM 1.5G 100 mW $cm^{-2}$. The AM 1.5G light source with a spectral mismatch factor of 1.01 was calibrated by the National Institute of Metrology. The intensity of the AM 1.5G spectra was calibrated by a certified standard silicon solar cell (SRC-2020, Enlitech) calibrated by the National Institute of Metrology. *J*−*V* curves were measured in the forward direction from −1.5 to 1.5 V, with scan step of 0.02 V and a dwell time of 5 ms. The cells were characterized under a temperature of 26-34 °C in a glove box filled with nitrogen. The area of the devices was determined by an optical microscope. The effective areas of the cells were 0.09 $cm^2$, respectively. The OSCs were measured by the aperture (0.06169 $cm^2$), and the *J-V* measurements have been performed with a mask covering all edges and the backside of the substrate to ensure the accuracy of the test. The device was encapsulated with UV curving adhesive, and the encapsulated devices were irradiated under a 365 nm UV lamp for 15 min. The encapsulated device was placed on a hot plate at 80˚C and intermittently tested by using a solar simulator along with AM 1.5G spectra in the glove box under a temperature of 26-34 °C. The dependence of the device performance on the light intensity was measured by tuning the light intensity via neutral density filters, the light intensity was calibrated by a standard Si solar cell. The external quantum efficiency (EQE) tests were carried out by using an integrated IPCE measurement system, namely QE-R3011 (Enli Technology Co. Ltd.). Photoinduced charge extraction by linearly increasing the voltage Photoinduced charge extraction by linearly increasing the voltage (photo-CELIV),

transient photovoltage (TPV), and transient short-circuit photocurrent (TPC) measurements were performed using the commercially available Paios system (FLUXiM AG, Switzerland). Sensitive EQE measurements were performed using a Vertex 70 (Bruker Optics). Electroluminescence (EL) quantum efficiency ($EQE_{EL}$) measurements were performed by applying external voltage/current sources through the devices (ELCT-3010, Enlitech). Single-Crystal data was collected at -173 [illegible] on a Bruker APEX II single-crystal X-ray diffractometer, Moradiation. The atomic force microscopy (AFM) images were obtained on a Nanoscope V AFM (Bruker) by tapping mode. GIWAXS measurements were conducted on a Xeuss 2.0 SAXS/WAXS system (Xenocs SA, France). with X-ray wavelength of 1.5418 Å. The film samples were irradiated at a fixed angle of 0.3°. The stability of the devices were measured by a Muti-channel Thin Film Photovoltaic Performance Decay Testing System (Model: PVLT-6001M-16A) with tracing the maximum output point. Hole and electron mobilities were measured by space charge limited current (SCLC) method, hole-and electron-only devices were fabricated by using the device structures of ITO/$MoO_3$/active layer/$MoO_3$/Ag and electron-only devices with a structure of ITO/ZnO/active layer/PFN-Br/Al, respectively. The mobility was calculated with the Mott−Gurney equation in the SCLC region: $J=9\varepsilon_0\varepsilon_r\mu V^2/8d^3$, where $J$ is the space charge limited current, $\varepsilon_0$ is the permittivity of free space, $\varepsilon_r$ is the relative permittivity of the material, $d$ is the thickness of the active layer and $V$ is the effective voltage. The effective voltage was obtained by subtracting the built-in voltage ($V_{bi}$) from the applied voltage ($V_{app}$), $V=V_{app}-V_{bi}$.

***DFT calculation***

The molecular geometries were optimized by Gaussian 09 with a functional of B3LYP and a basis set of 6-31G(d,p).[4] The long alkyl chains were replaced by methyl for saving computation time. The ESP analysis was carried out by a wavefunction analysis tool Multiwfn.[5]

## SUPPLEMENTARY FIGURES

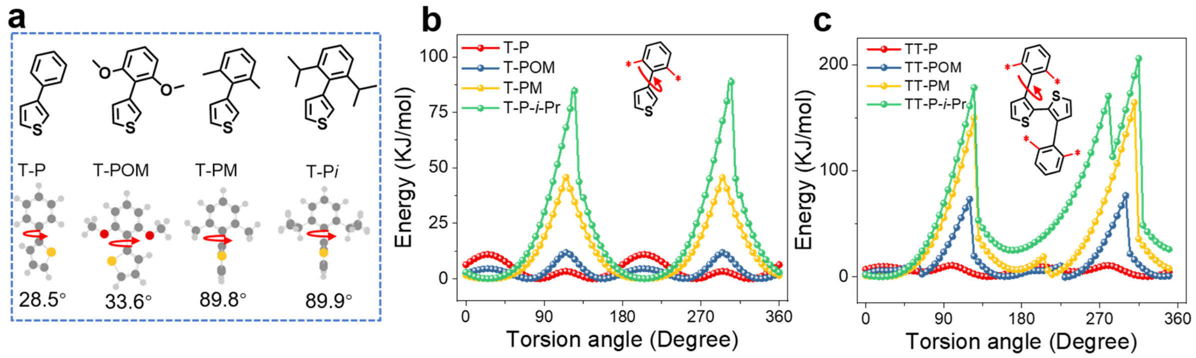


**Supplementary Figure 1. The density functional theory (DFT) calculation results. a** Chemical structures and DFT-calculated torsion angles of the optimal geometries for several thiophene units at the B3LYP / 6-31G (d, p) level. **b** Torsional energy profiles between thiophene and benzene ring for thiophene segments. **c** Torsional energy profiles between thiophene and benzene ring for several bithiophene segments.

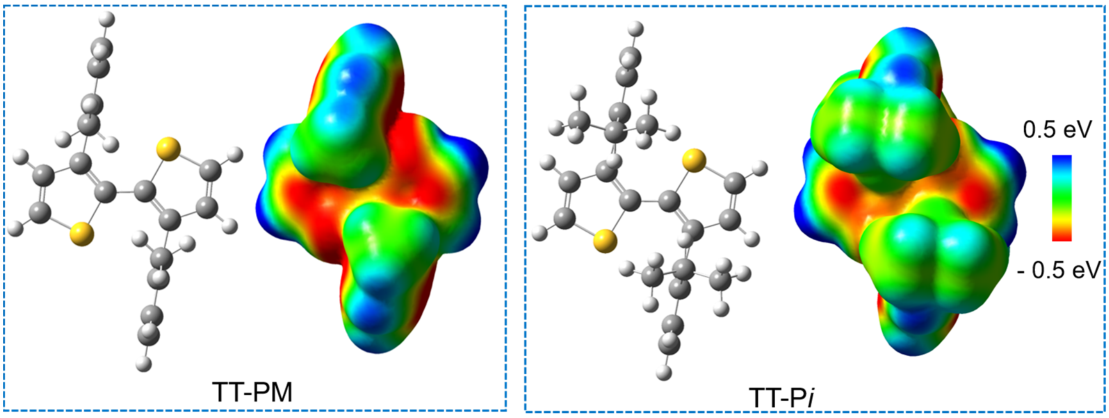


**Supplementary Figure 2.** The ESP distributions of the TT-PM and TT-P*i* units.

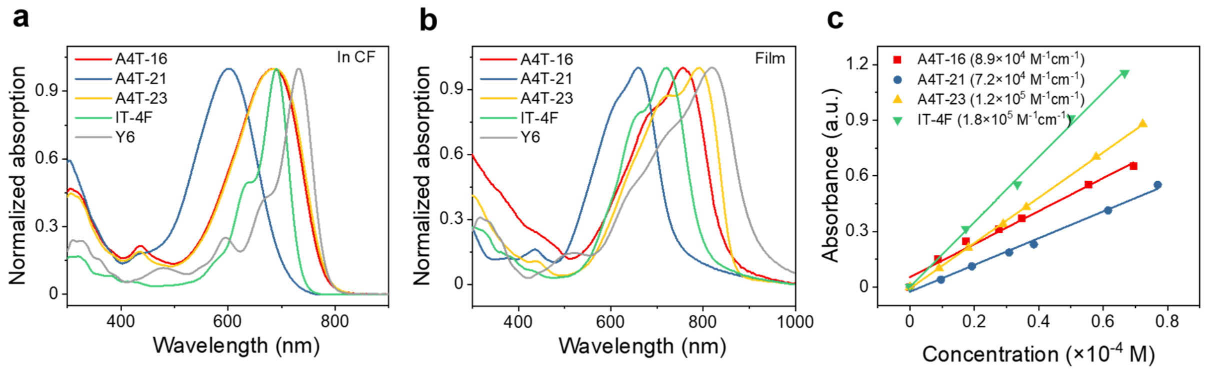


**Supplementary Figure 3. Photophysical properties of NFAs.** UV−vis spectra of A4T-16, A4T-21, A4T-23, IT-4F and Y6 **a** in CF solution, **b** in solid films, **c** absorption coefficients in CF solution.

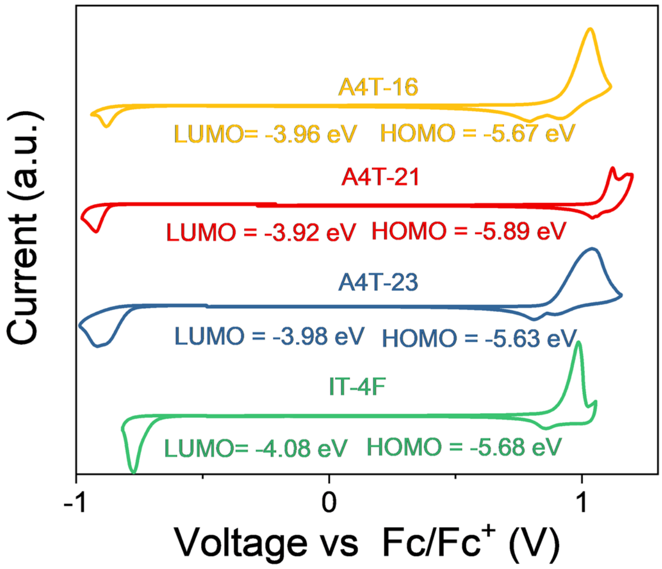


**Supplementary Figure 4.** Electrochemical Cyclic voltammogram for A4T-16, A4T-21, A4T-23, and IT-4F in $CH_3CN$/0.1 M $[n^{-}Bu_4N]^{+}[PF_6]^{-}$ at a scan rate of 50 mV $s^{-1}$.

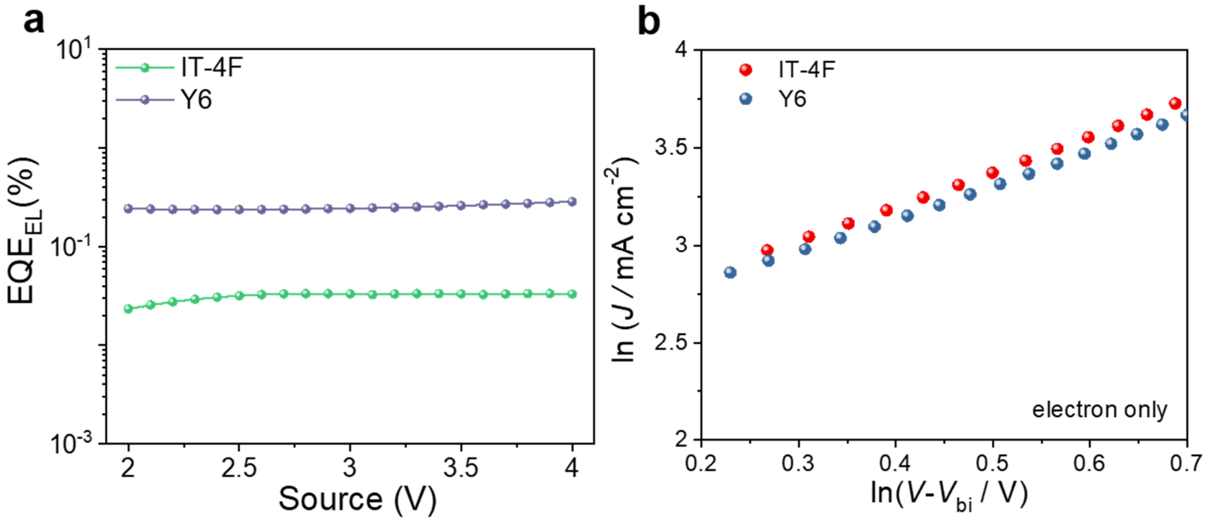


**Supplementary Figure 5. a** $EQE_{EL}$ curves, **b** electron mobilities of IT-4F and Y6.

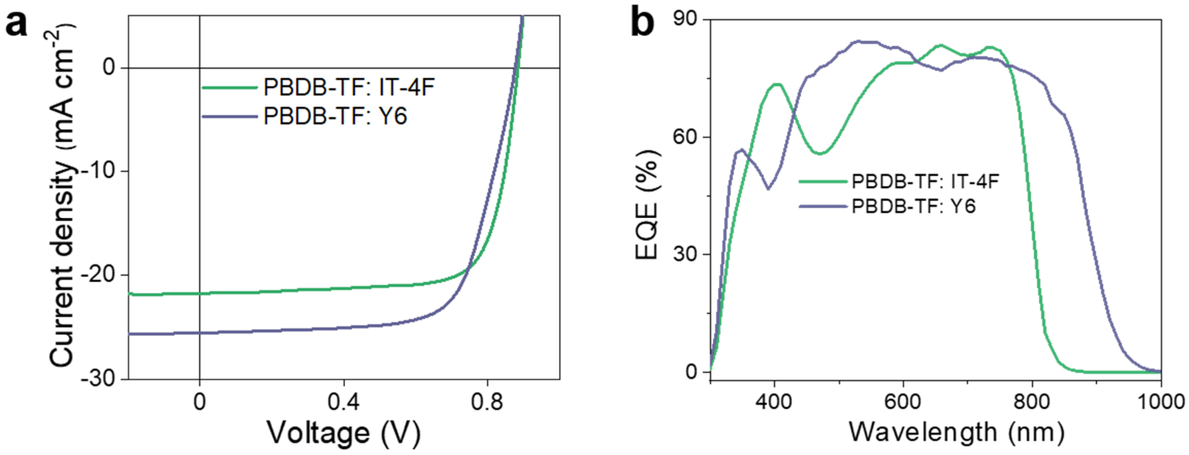


**Supplementary Figure 6. Photovoltaic properties of the OPV cells. a** *J*-*V* curves, **b** EQE curves of PBDB-TF:IT-4F-, PBDB-TF: Y6-based cells.

**Appendix: Summary of the Report**
**Report No.: GXgf2021-11598**
**Client: INSTITUTE OF CHEMISTRY, CAS**
**Sample: Organic Solar Cells**
**Type/Model: Organic Solar Cells**
**DUT S/N: 2-M1-F-1**
**Manufacturer: INSTITUTE OF CHEMISTRY, CAS**
**Date of Test: 06/10/2021**
**Temperature Sensor/Control System: None**
**Environmental conditions: (24.3±1) C°, RH (51.4±2) %**
**Mask: An aperture area of 6.169 mm² (M1, Certificate No.: CDjc2019-6589)**

The test has been conducted by the PV Metrology Lab of NIM (National Institute of Metrology, China). Measurement of irradiance intensity and all other measurements are traceable to the International System of Units (SI). Data in this report apply only at the time of the test for the sample. For more details, please refer to the text of the report.

Forward Scan：

| Area ($mm^2$) | $I_{sc}$(mA) | $V_{oc}$(V) | $P_{max}$(mW) |
|---|---|---|---|
| 6.169 | 1.318 | 0.879 | 0.916 |
| $I_{max}$(mA) | $V_{max}$(V) | FF (%) | $\eta$ (%) |
| 1.205 | 0.760 | 79.1 | 14.8 |

**I-V Characterization Methods:**
JJF 1622-2017: Calibration Specification of Solar Cells：Photoelectric Properties
**Secondary Reference Cell:**
Device S/N: 81#; Device Material: Mono-Si
**Solar Simulator:**
Classification: AAA (Double-light source: Xeon and Halogen);
Total irradiance: 1000 W/m² based on $I_{sc}$ of the above Secondary Reference Cell.

**Supplementary Figure 7.** The photovoltaic certification of the PBDB-TF:A4T-16-based cell by National Institute of Metrology, China**.**

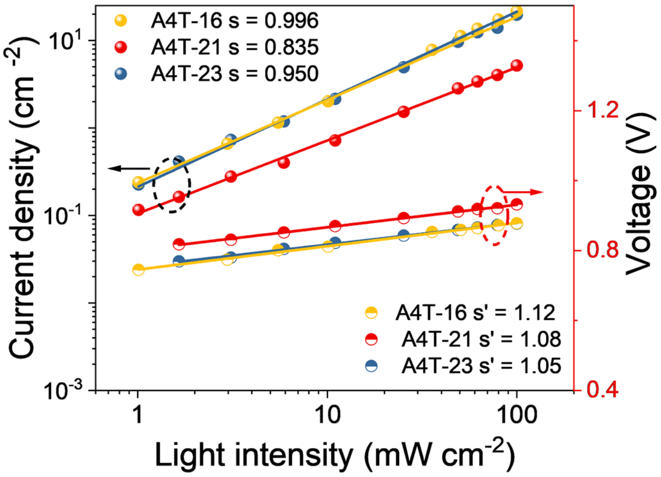


**Supplementary Figure 8.** The light-intensity ($P_{light}$) dependence of $J_{SC}$ and $V_{OC}$ of the corresponding devices.

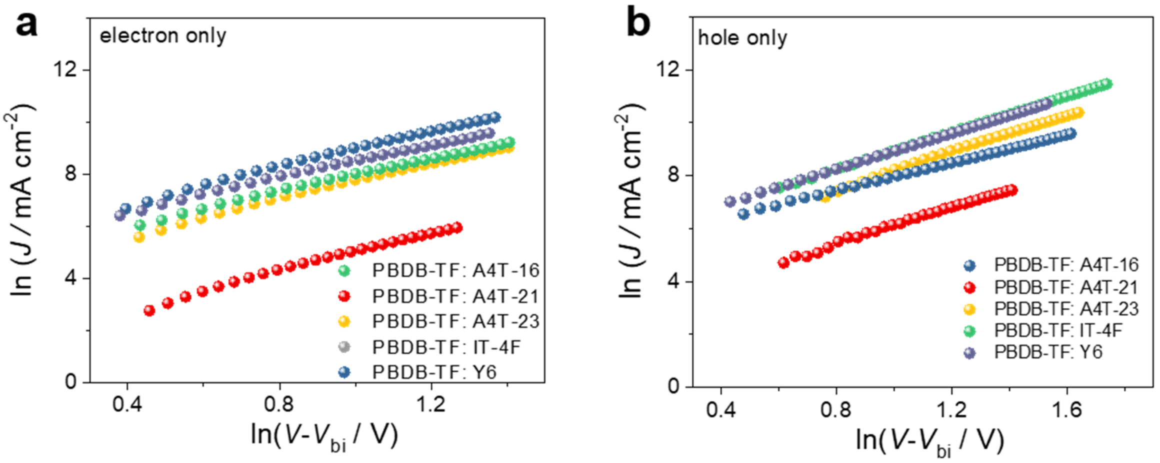


**Supplementary Figure 9.** The SCLC of these blends. **a** hole mobilities and **b** electron mobilities of blend films based on A4T-16, A4T-21, A4T-23, IT-4F, and Y6.

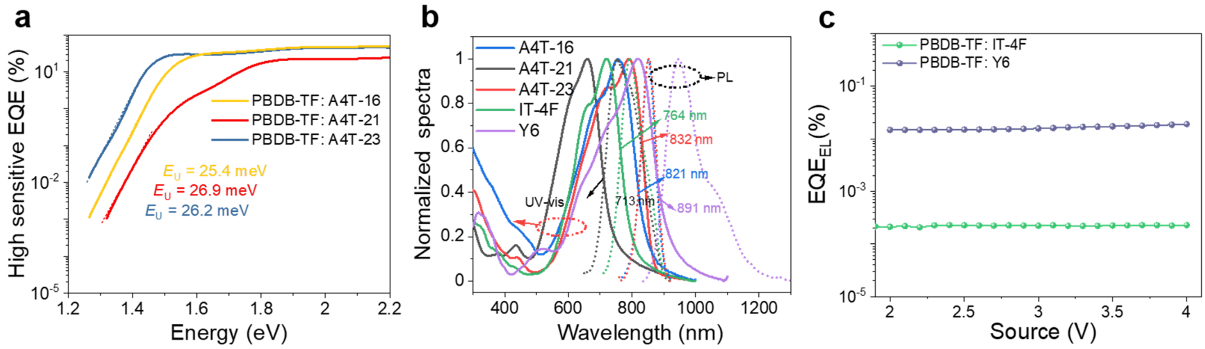


**Supplementary Figure 10. a** s-EQE spectra of A4T-16, A4T-21, and A4T-23-based cells. **b** Optical gap determined by the crossing point between the normalized emission and absorption spectra. **c** $EQE_{EL}$ curves of PBDB-TF: IT-4F and PBDB-TF: Y6-based cells.

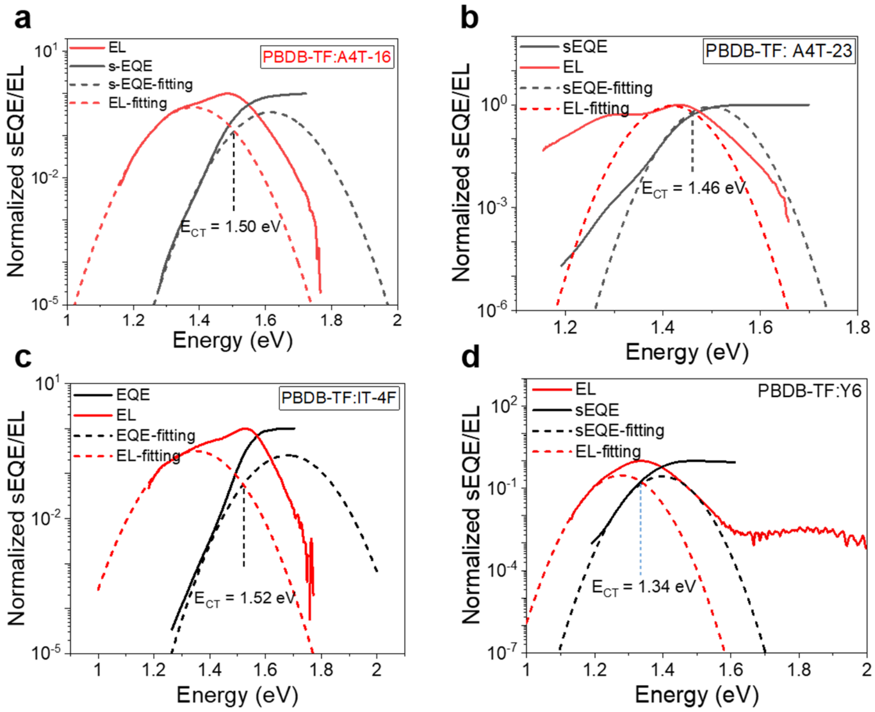


**Supplementary Figure 11.** s-EQE and EL spectra of **a** PBDB-TF: A4T-16-, **b** PBDB-TF: A4T-23-, **c** PBDB-TF: IT-4F-, **d** PBDB-TF: Y6-based devices.

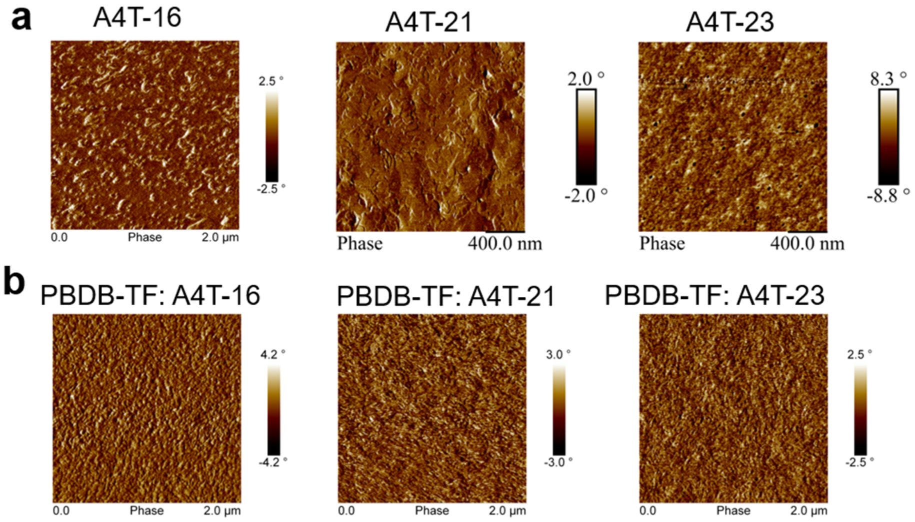


**Supplementary Figure 12.** AFM phase images of the pure neat films of the three NFAs and their blend films.

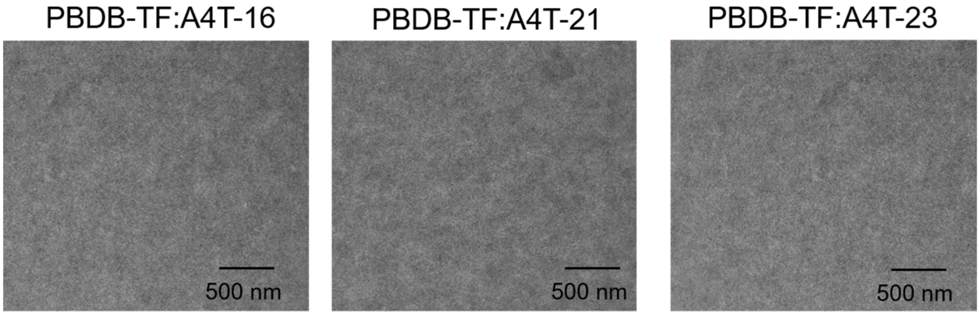


**Supplementary Figure 13.** TEM images of the PBDB-TF:A4T-16, PBDB-TF:A4T-21 and PBDB-TF:A4T-23 blend films.

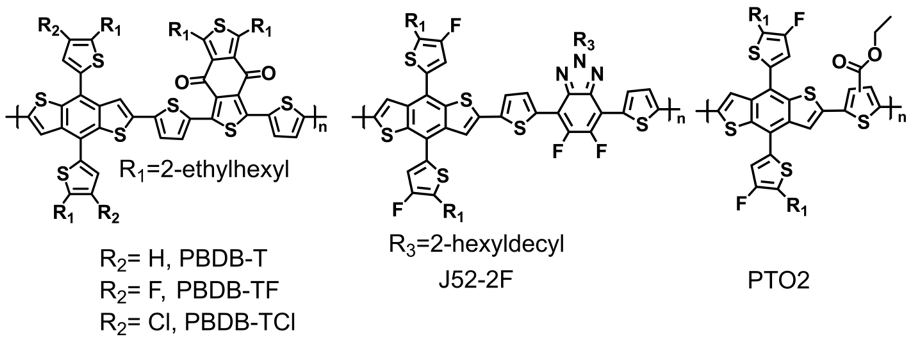


**Supplementary Figure 14.** Chemical structures of PBDB-T, PBDB-TF, J52-2F, PTO2, and PBDB-TCl.

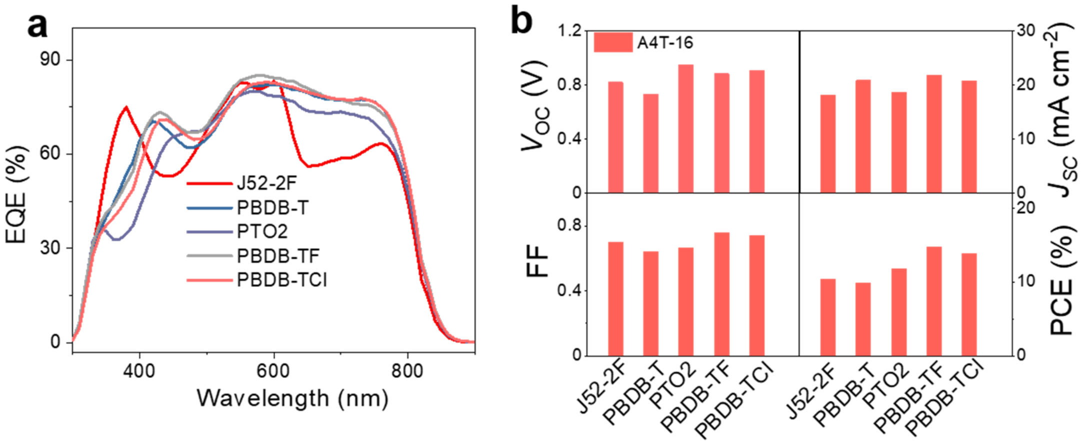


**Supplementary Figure 15. Photovoltaic properties of the OPV cells. a** EQE curves, **b** $V_{OC}$s, $J_{SC}$s, FFs, and PCEs of polymers: A4T-16-based cells.

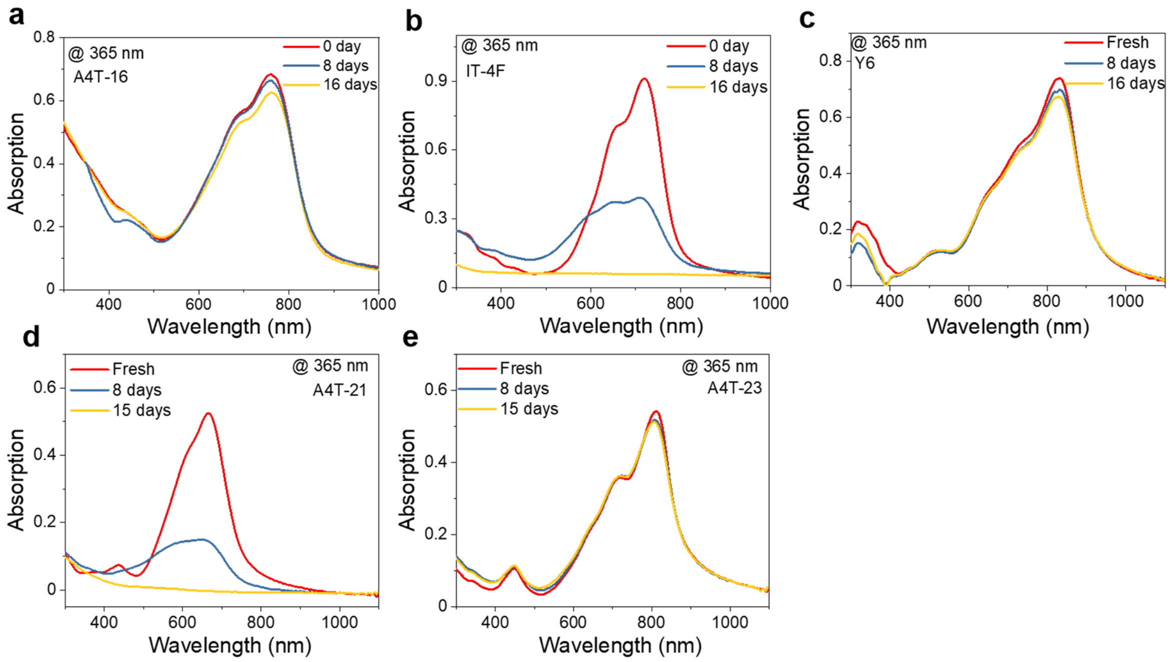


**Supplementary Figure 16.** The absorption spectra for A4T-16, IT-4F, Y6, A4T-21 and A4T-23 under the illumination of UV-light (365 nm) for 16 days.

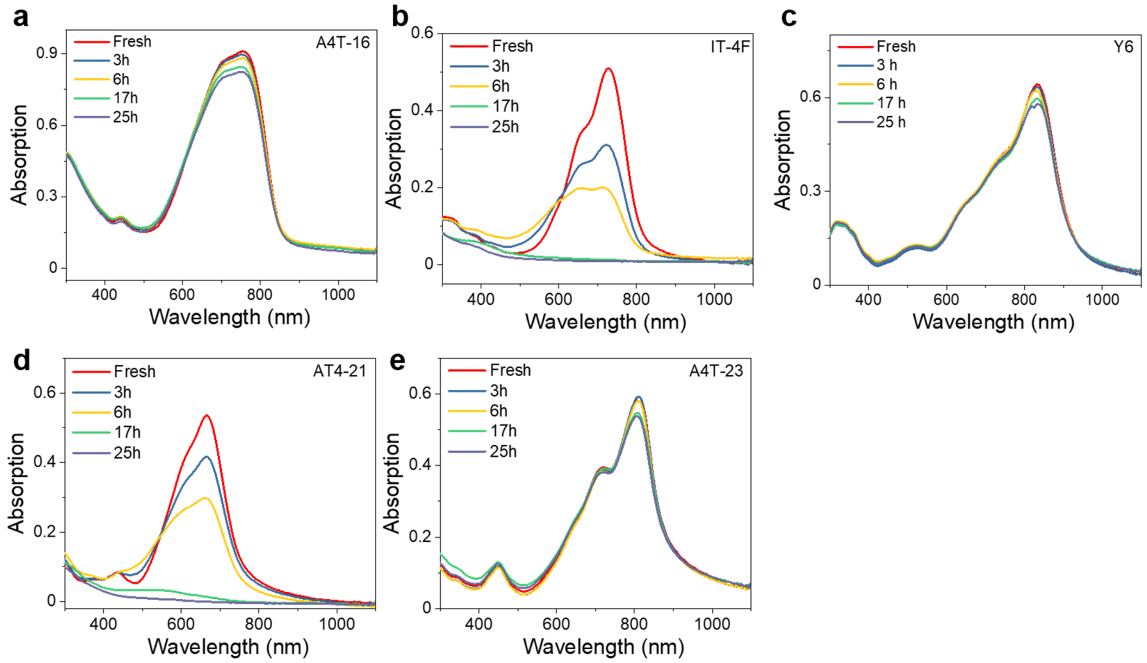


**Supplementary Figure 17.** The absorption spectra for A4T-16, IT-4F, Y6, A4T-21 and A4T-23 under the continuous 1-sun-equivalent illumination.

## SUPPLEMENTARY TABLES

**Supplementary Table 1**. Optical and electrochemical properties of the NFAs.

| Acceptor | $\lambda_{max}$ (nm) [a] | $\lambda_{max}$ (nm) [b] | $E_g^{opt}$ (eV) | HOMO (eV) | LUMO (eV) |
|---|---|---|---|---|---|
| A4T-16 | 686 | 756 | 1.43 | -5.67 | -3.96 |
| A4T-21 | 601 | 660 | 1.66 | -5.89 | -3.92 |
| A4T-23 | 686 | 800 | 1.45 | -5.63 | -3.98 |
| IT-4F | 690 | 720 | 1.53 | -5.68 | -4.08 |
| Y6 | 733 | 819 | 1.34 | -5.65 | -4.10 |

[a] in solution, [b] thin film.

**Supplementary Table 2**. Mobilities of NFAs and the OSCs based on PBDB-TF: NFAs.

| Materials | $\mu_h$ [cm$^2$ V$^{-1}$ s$^{-1}$] | $\mu_e$ [cm$^2$ V$^{-1}$ s$^{-1}$] | $\mu_h/\mu_e$ | $\mu_{celiv}$ [cm$^2$ V$^{-1}$ s$^{-1}$] |
|---|---|---|---|---|
| A4T-16 | - | $5.5 \times 10^{-5}$ | - | – |
| A4T-21 | - | $1.9 \times 10^{-5}$ | - | – |
| A4T-23 | - | $2.7 \times 10^{-5}$ | - | – |
| IT-4F | - | $5.9 \times 10^{-5}$ | - | – |
| Y6 | - | $5.4 \times 10^{-5}$ | - | – |
| PBDB-TF: A4T-21 | $2.5 \times 10^{-5}$ | $1.2 \times 10^{-5}$ | 2.1 | $1.9 \times 10^{-4}$ |
| PBDB-TF: A4T-23 | $2.7 \times 10^{-4}$ | $2.5 \times 10^{-4}$ | 1.1 | $3.0 \times 10^{-4}$ |
| PBDB-TF: A4T-16 | $3.0 \times 10^{-4}$ | $2.9 \times 10^{-4}$ | 1.0 | $2.4 \times 10^{-4}$ |
| PBDB-TF: IT-4F | $6.0 \times 10^{-4}$ | $5.4 \times 10^{-4}$ | 1.1 | $2.6 \times 10^{-4}$ |
| PBDB-TF: Y6 | $6.5 \times 10^{-4}$ | $6.3 \times 10^{-4}$ | 1.0 | $3.2 \times 10^{-4}$ |

**Supplementary Table 3.** Photovoltaic parameters of the PBDB-TF: IT-4F - and PBDB-TF: Y6-based cells.

| Blends | $V_{OC}$ [V] | $J_{SC}$ [mA cm$^{-2}$] [a)] | FF | PCE [b)] (%) max (average)] |
|---|---|---|---|---|
| PBDB-TF: IT-4F | 0.852 | 21.3 (20.2) | 0.757 | 13.7 (13.2 ± 0.4) |
| PBDB-TF: Y6 | 0.841 | 25.5 (25.3) | 0.737 | 15.8 (15.4 ± 0.4) |

a) Calculated by EQE integration; b) Average PCE of ten devices.

**Supplementary Table 4**. Hansen solubility parameters of PBDB-TF, A4T-16, A4T-21 and A4T-23.

| HSPs | PBDB-TF | A4T-16 | A4T-21 | A4T-23 |
|---|---|---|---|---|
| $\delta_d$ | 18.9 | 18.34 | 18.13 | 18.65 |
| $\delta_p$ | 4.4 | 2.19 | 2.49 | 2.31 |
| $\delta_h$ | 6.4 | 4.43 | 4.73 | 4.55 |

Note: Three components {$\delta_d$, $\delta_p$, $\delta_h$} representing the dispersive, polar, and hydrogen bonding forces, respectively.

**Supplementary Table 5.** χ parameters of the blend films.

| Blends | PBDB-TF:A4T-16 | PBDB-TF:A4T-21 | PBDB-TF:A4T-23 |
|---|---|---|---|
| χ | 0.52 | 0.43 | 0.41 |

**Supplementary Table 6.** Total and detailed voltage losses of OSCs.

| Blend | $E_g$ (eV)[a)] | $V_{OC}$ | $V_{loss}$ (V) | $E_{CT}$ (eV) | $\Delta E_{CT}$ (eV) | $\Delta E_{rad}$ (V) | $\Delta E_{non\text{-}rad}$ (V) | $EQE_{EL}$ |
|---|---|---|---|---|---|---|---|---|
| PBDB-TF: A4T-16 | 1.51 | 0.876 | 0.634 | 1.50 | 0.01 | 0.326 | 0.298 | $1.08\times10^{-5}$ |
| PBDB-TF: A4T-21 | 1.74 | 0.936 | 0.804 | 1.52 | 0.22 | 0.329 | 0.255 | $5.29\times10^{-5}$ |
| PBDB-TF: A4T-23 | 1.49 | 0.870 | 0.620 | 1.46 | 0.03 | 0.363 | 0.227 | $1.56\times10^{-4}$ |
| PBDB-TF: Y6 | 1.39 | 0.841 | 0.549 | 1.34 | 0.05 | 0.273 | 0.226 | $1.58\times10^{-4}$ |
| PBDB-TF: IT-4F | 1.63 | 0.852 | 0.778 | 1.52 | 0.11 | 0.332 | 0.336 | $2.30\times10^{-6}$ |

a) The $E_g$ values are determined from the crossing point between the normalized emission and absorption spectra of the acceptor neat films.

**Supplementary Table 7** Number of synthetic steps (NSS), reciprocal yield (RY), number of operation units for the isolation/purification (NUO), number of column chromatographies for the isolation/purification (NCC), number of hazardous chemicals (NHC), synthetic complexity index (SC), figure-of-merit (FoM) for five acceptors

| Acceptors | NSS [a)] | RY | NCC | NUO | NHC | SC index% | PCE (%) | FoM |
|---|---|---|---|---|---|---|---|---|
| A4T-16 | 8 | 2.34 | 4 | 24 | 46 | 47.03 | 15.2 | 32.3 |
| A4T-21 | 8 | 2.49 | 4 | 24 | 46 | 47.05 | 1.57 | 3.34 |
| A4T-23 | 8 | 2.38 | 4 | 24 | 46 | 47.03 | 10.4 | 22.1 |
| IT-4F | 11 | 11.1 | 6 | 39 | 66 | 86.13 | 13.7 | 15.9 |
| Y6 | 12 | 19.6 | 7 | 42 | 73 | 100 | 15.8 | 15.8 |

a) The synthesis of the terminal unit is not included in the calculations. FoM is defined as followed: FoM = PCE/SC.

**Supplementary Table 8** Crystal data and structure refinement for 2074697.

| Identification code | 2074697 |
|---|---|
| Empirical formula | $C_{88}H_{90}F_4N_4O_2S_4$ |

| | |
|---|---|
| Formula weight | 1439.87 |
| Temperature/K | 170.00(12) |
| Crystal system | monoclinic |
| Space group | C2/c |
| a/Å | 21.0980(4) |
| b/Å | 29.4875(4) |
| c/Å | 14.7690(4) |
| α/° | 90 |
| β/° | 106.593(3) |
| γ/° | 90 |
| Volume/Å$^3$ | 8805.6(3) |
| Z | 4 |
| $\rho_{calc}$g/cm$^3$ | 1.086 |
| μ/mm$^{-1}$ | 1.415 |
| F(000) | 3048.0 |
| Crystal size/mm$^3$ | 0.4 × 0.28 × 0.25 |
| Radiation | Cu Kα (λ = 1.54184) |
| 2θ range for data collection/° | 5.3 to 151.912 |
| Index ranges | $-26 \leq h \leq 26, -36 \leq k \leq 36, -18 \leq l \leq 18$ |
| Reflections collected | 57670 |
| Independent reflections | 8845 [$R_{int}$ = 0.0357, $R_{sigma}$ = 0.0168] |
| Data/restraints/parameters | 8845/205/508 |
| Goodness-of-fit on F$^2$ | 2.327 |
| Final R indexes [I>=2σ (I)] | $R_1$ = 0.0978, w$R_2$ = 0.2976 |
| Final R indexes [all data] | $R_1$ = 0.1091, w$R_2$ = 0.3060 |
| Largest diff. peak/hole / e Å$^{-3}$ | 0.75/-0.38 |

## SUPPLEMENTARY NMR FIGURES

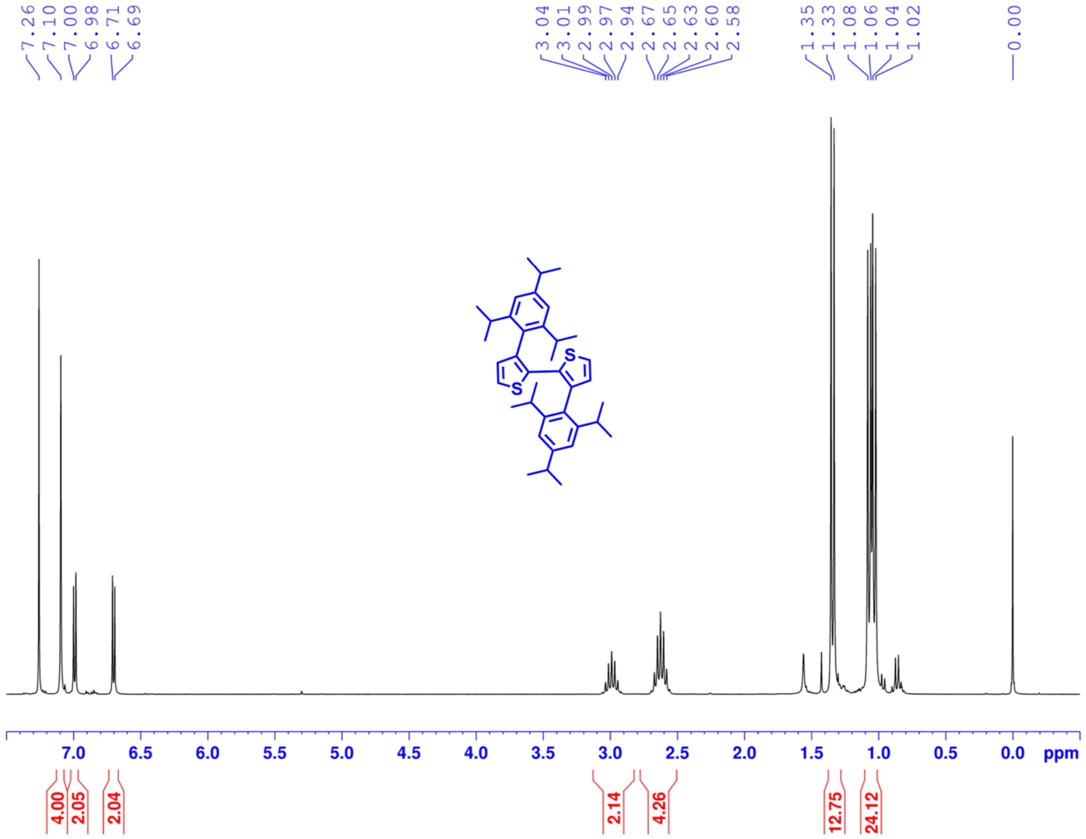


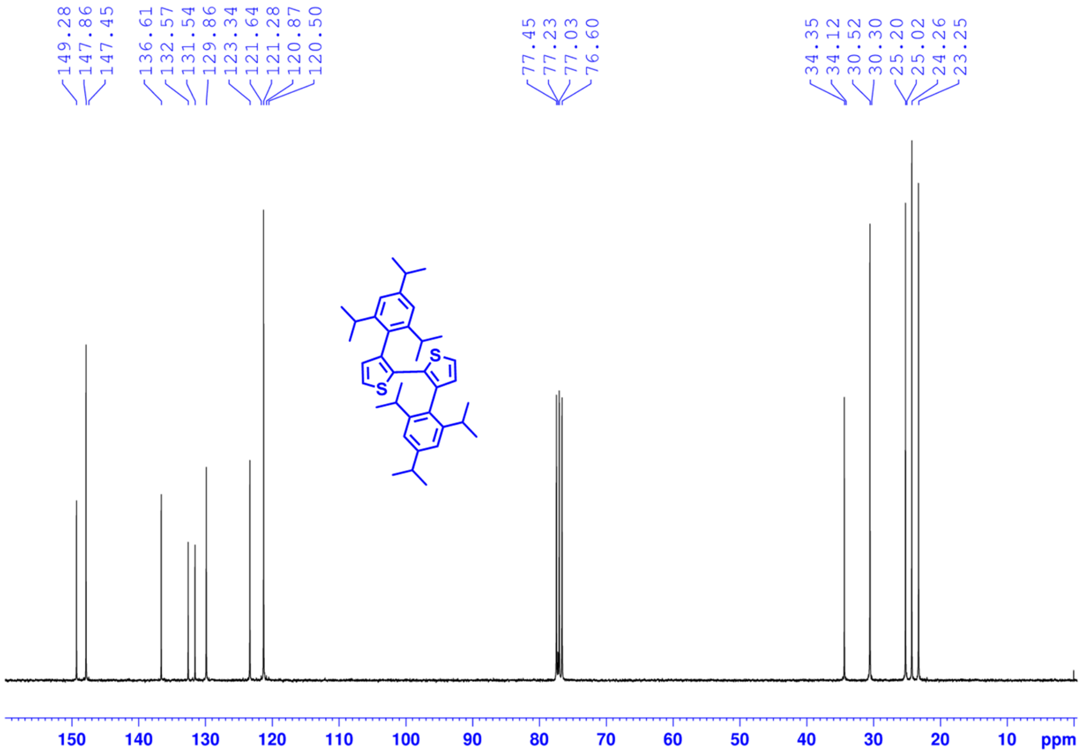

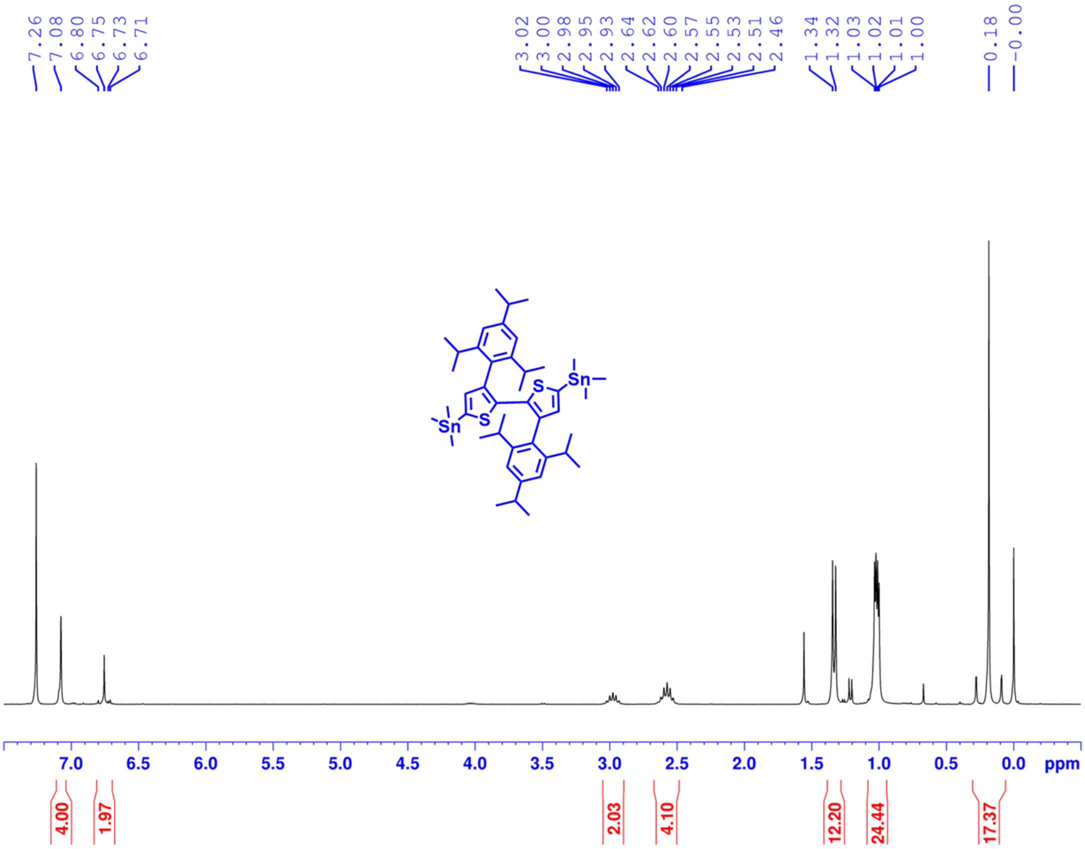
7.26 7.08 6.80 6.75 6.73 6.71
3.02 3.00 2.98 2.95 2.93 2.64 2.62 2.60 2.57 2.55 2.53 2.51 2.46
1.34 1.32 1.03 1.02 1.01 1.00
0.18 -0.00
4.00 1.97 2.03 4.10 12.20 24.44 17.37
ppm

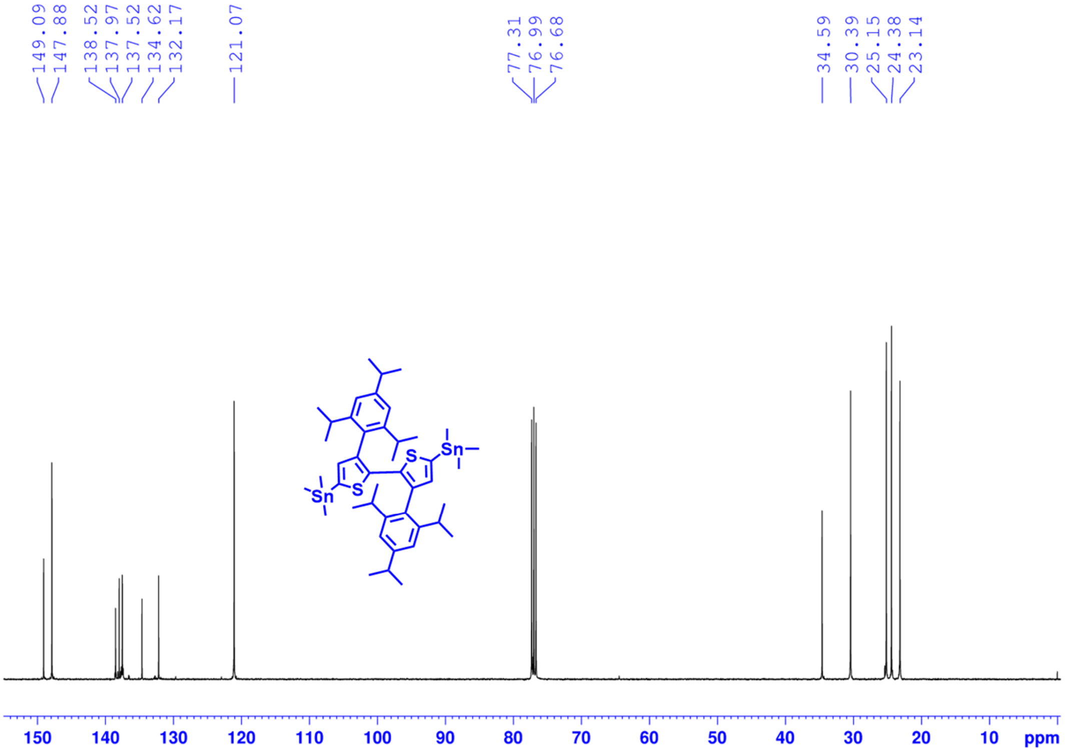
149.09 147.88 138.52 137.97 137.52 134.62 132.17 121.07
77.31 76.99 76.68
34.59 30.39 25.15 24.38 23.14
ppm

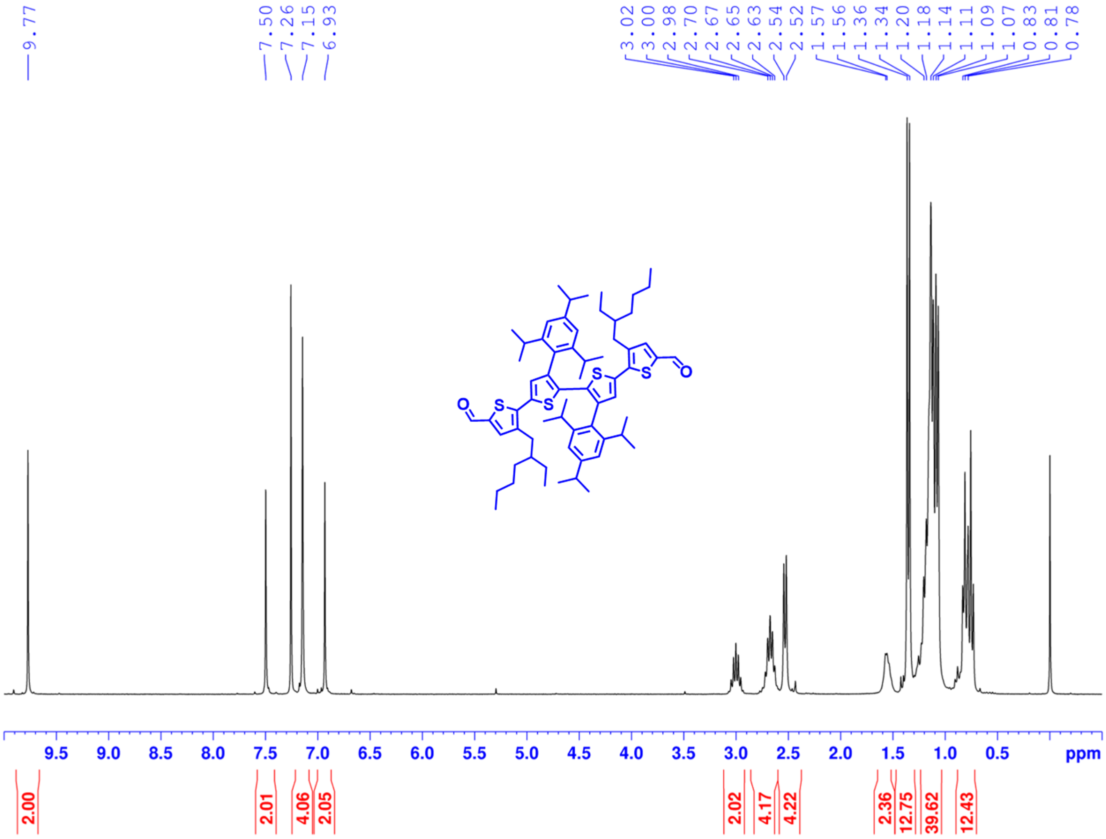
9.77
7.50
7.26
7.15
6.93
3.02 3.00 2.98 2.70 2.67 2.65 2.63 2.54 2.52 1.57 1.56 1.36 1.34 1.20 1.18 1.14 1.11 1.09 1.07 0.83 0.81 0.78
S
O
9.5 9.0 8.5 8.0 7.5 7.0 6.5 6.0 5.5 5.0 4.5 4.0 3.5 3.0 2.5 2.0 1.5 1.0 0.5 ppm
2.00
2.01
4.06
2.05
2.02
4.17
4.22
2.36
12.75
39.62
12.43

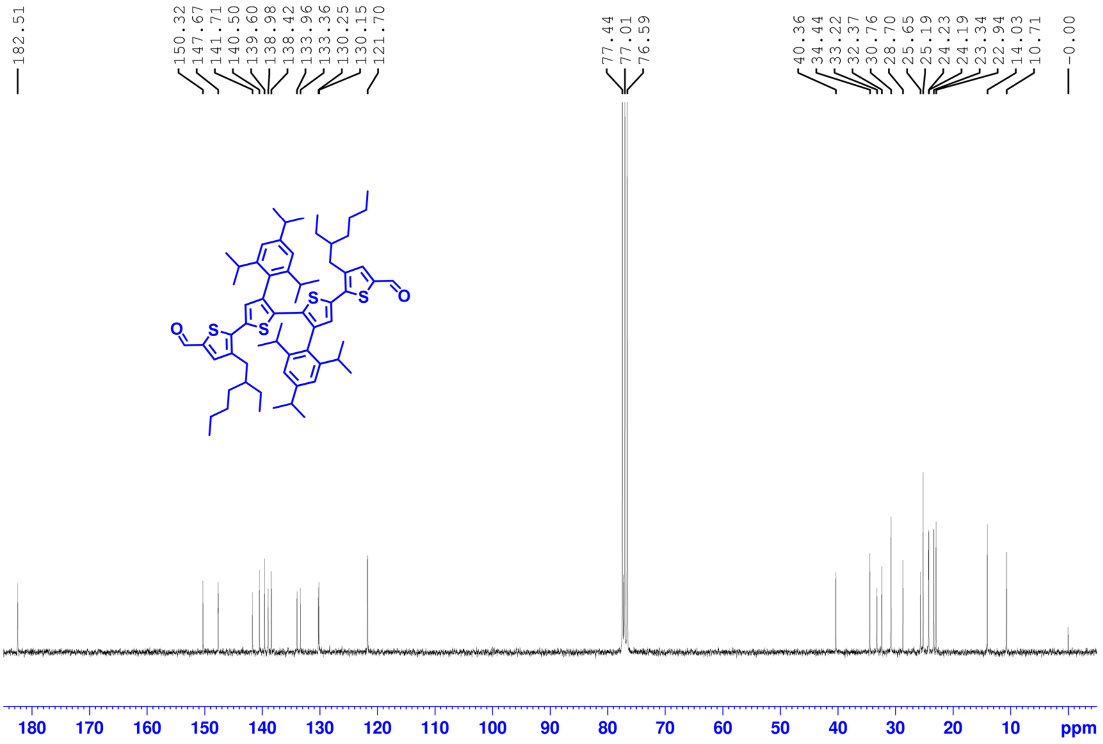
182.51
150.32 147.67 141.71 140.50 139.60 138.98 138.42 133.96 133.36 130.25 130.15 121.70
77.44 77.01 76.59
40.36 34.44 33.22 32.37 30.76 28.70 25.65 25.19 24.23 24.19 23.34 22.94 14.03 10.71
-0.00
S
O
180 170 160 150 140 130 120 110 100 90 80 70 60 50 40 30 20 10 ppm

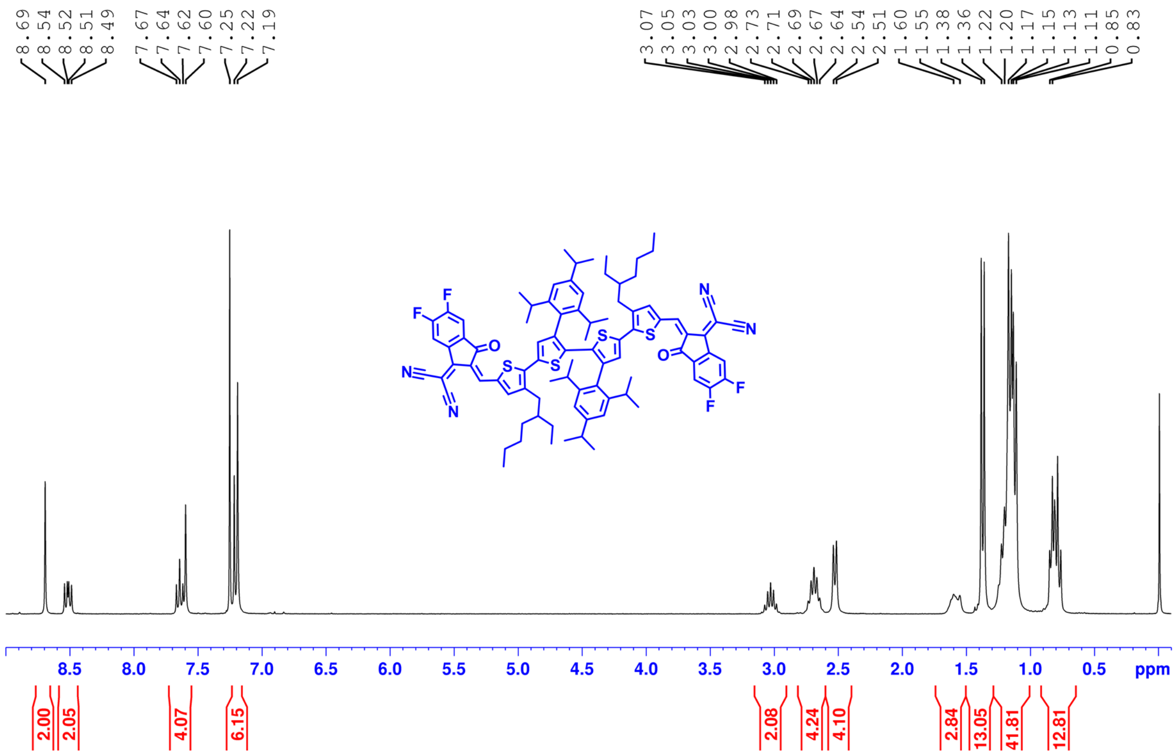
8.69
8.54
8.52
8.51
8.49
7.67
7.64
7.62
7.60
7.25
7.22
7.19
3.07
3.05
3.03
3.00
2.98
2.73
2.71
2.69
2.67
2.64
2.54
2.51
1.60
1.55
1.38
1.36
1.22
1.20
1.17
1.15
1.13
1.11
0.85
0.83
8.5 8.0 7.5 7.0 6.5 6.0 5.5 5.0 4.5 4.0 3.5 3.0 2.5 2.0 1.5 1.0 0.5 ppm
2.00
2.05
4.07
6.15
2.08
4.24
4.10
2.84
13.05
41.81
12.81

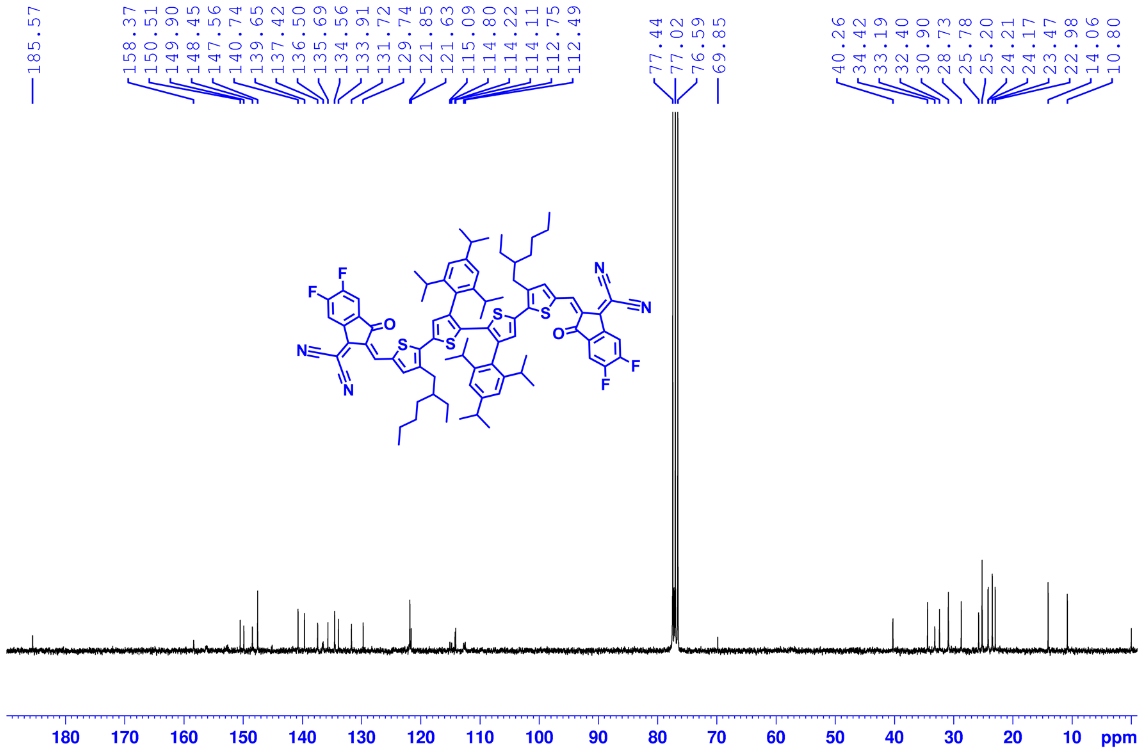
185.57
158.37
150.51
149.90
148.45
147.56
140.74
139.65
137.42
136.50
135.69
134.56
133.91
131.72
129.74
121.85
121.63
115.09
114.80
114.22
114.11
112.75
112.49
77.44
77.02
76.59
69.85
40.26
34.42
33.19
32.40
30.90
28.73
25.78
25.20
24.21
24.17
23.47
22.98
14.06
10.80
180 170 160 150 140 130 120 110 100 90 80 70 60 50 40 30 20 10 ppm

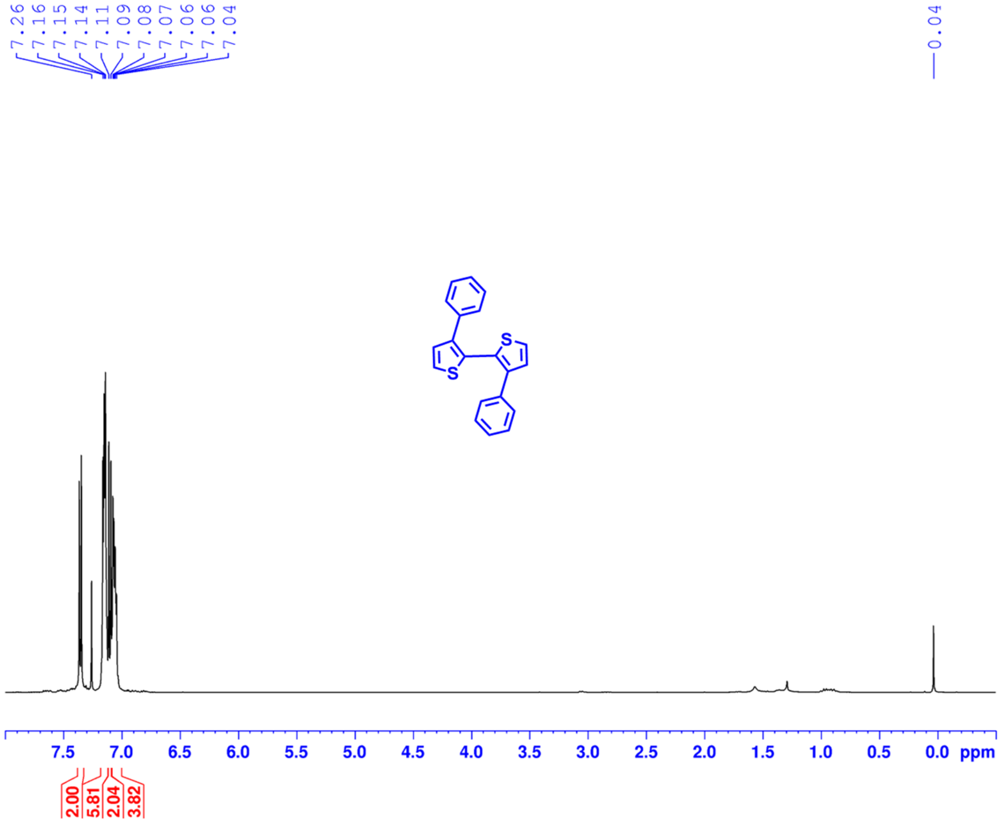
7.26
7.16
7.15
7.14
7.11
7.09
7.08
7.07
7.06
7.06
7.04
0.04
S
S
7.5 7.0 6.5 6.0 5.5 5.0 4.5 4.0 3.5 3.0 2.5 2.0 1.5 1.0 0.5 0.0 ppm
2.00
5.81
2.04
3.82

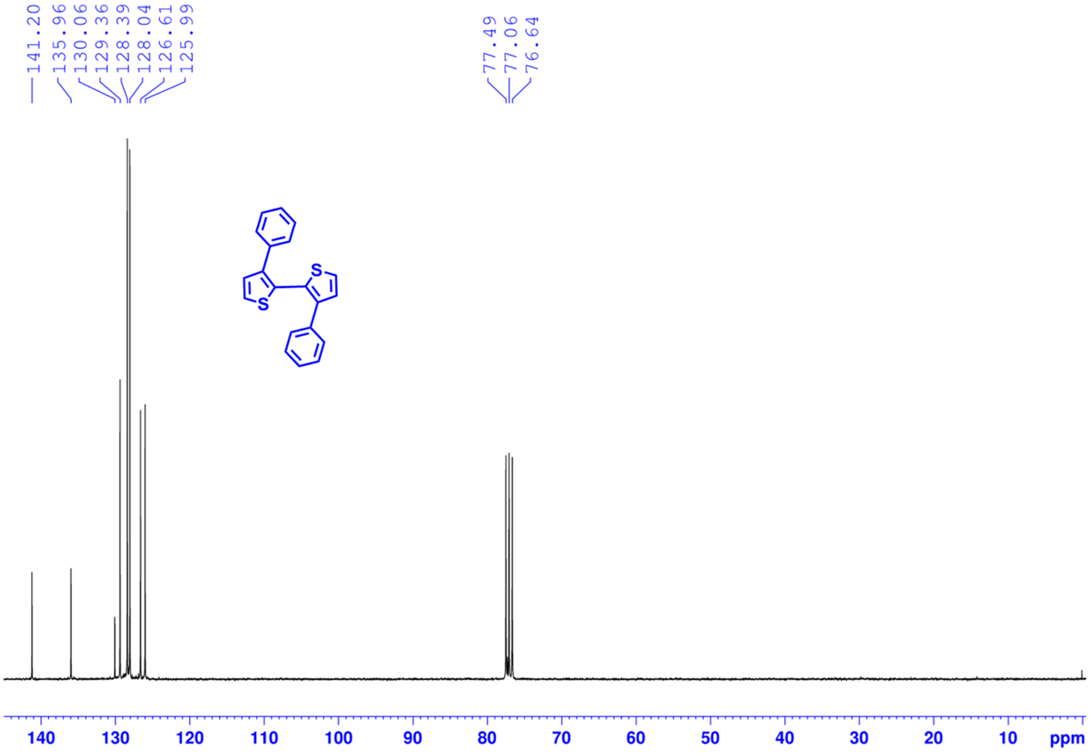
141.20
135.96
130.06
129.36
128.39
128.04
126.61
125.99
77.49
77.06
76.64
S
S
140 130 120 110 100 90 80 70 60 50 40 30 20 10 ppm

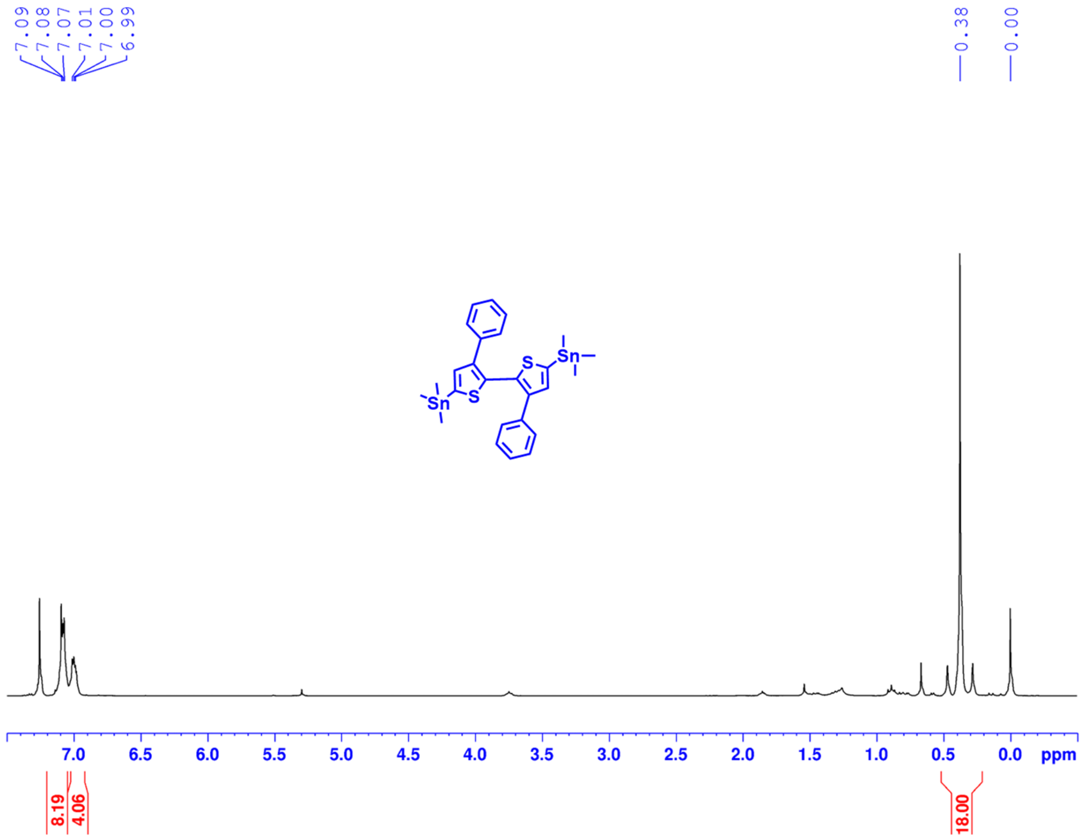
7.09
7.08
7.07
7.01
7.00
6.99
0.38
0.00
Sn
S
7.0
6.5
6.0
5.5
5.0
4.5
4.0
3.5
3.0
2.5
2.0
1.5
1.0
0.5
0.0
ppm
8.19
4.06
18.00

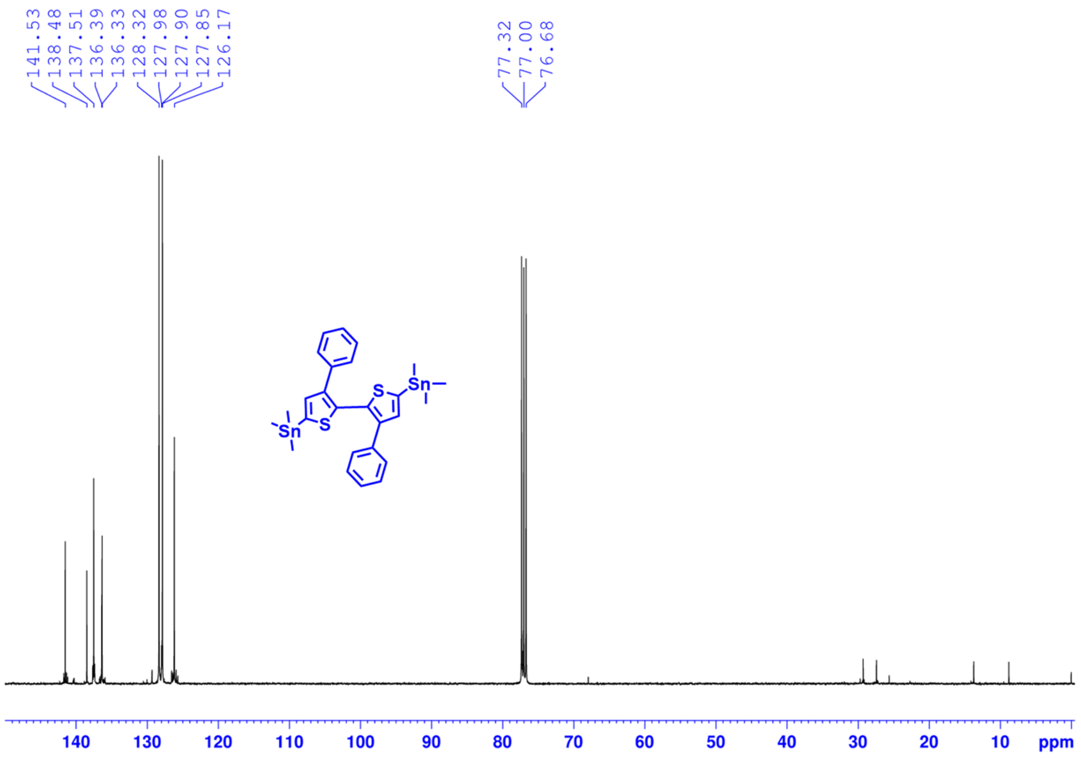
141.53
138.48
137.51
136.39
136.33
128.32
127.98
127.90
127.85
126.17
77.32
77.00
76.68
Sn
S
140
130
120
110
100
90
80
70
60
50
40
30
20
10
ppm

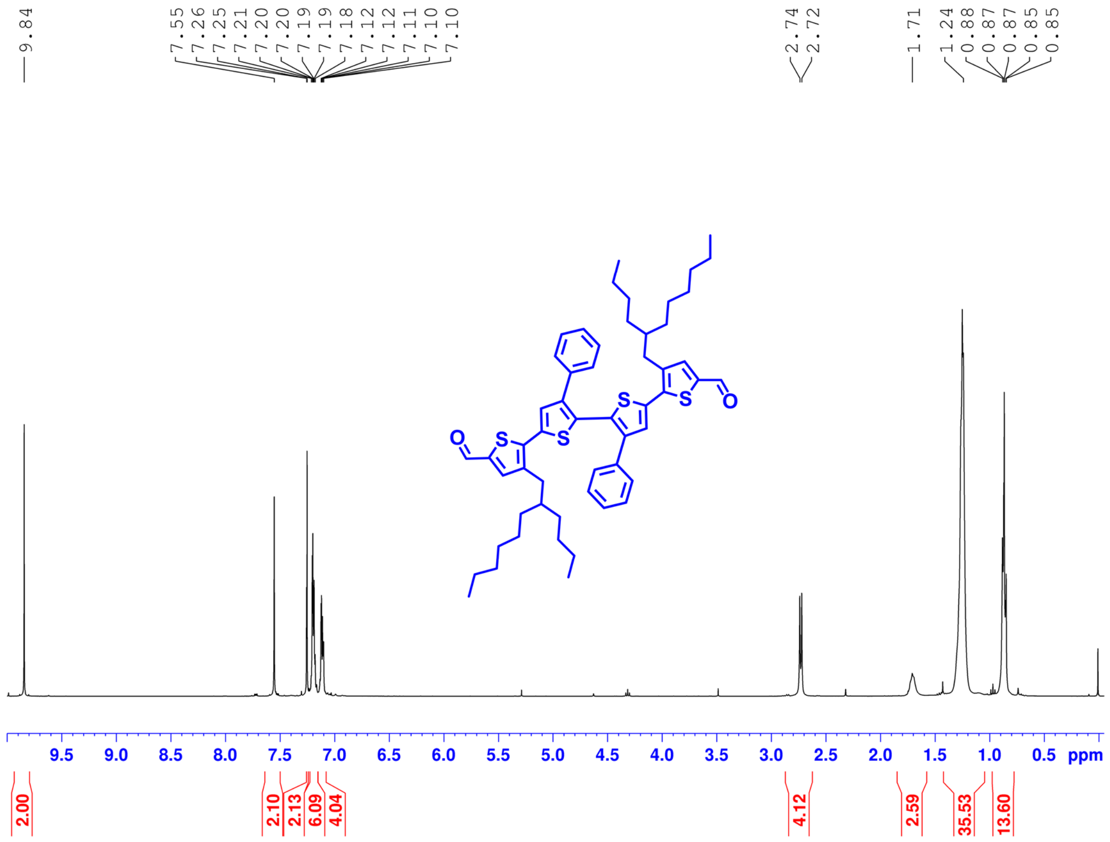
9.84
7.55 7.26 7.25 7.21 7.20 7.20 7.19 7.19 7.18 7.12 7.12 7.11 7.10 7.10
2.74 2.72
1.71 1.24 0.88 0.87 0.87 0.85 0.85
2.00 2.10 2.13 6.09 4.04 4.12 2.59 35.53 13.60
ppm

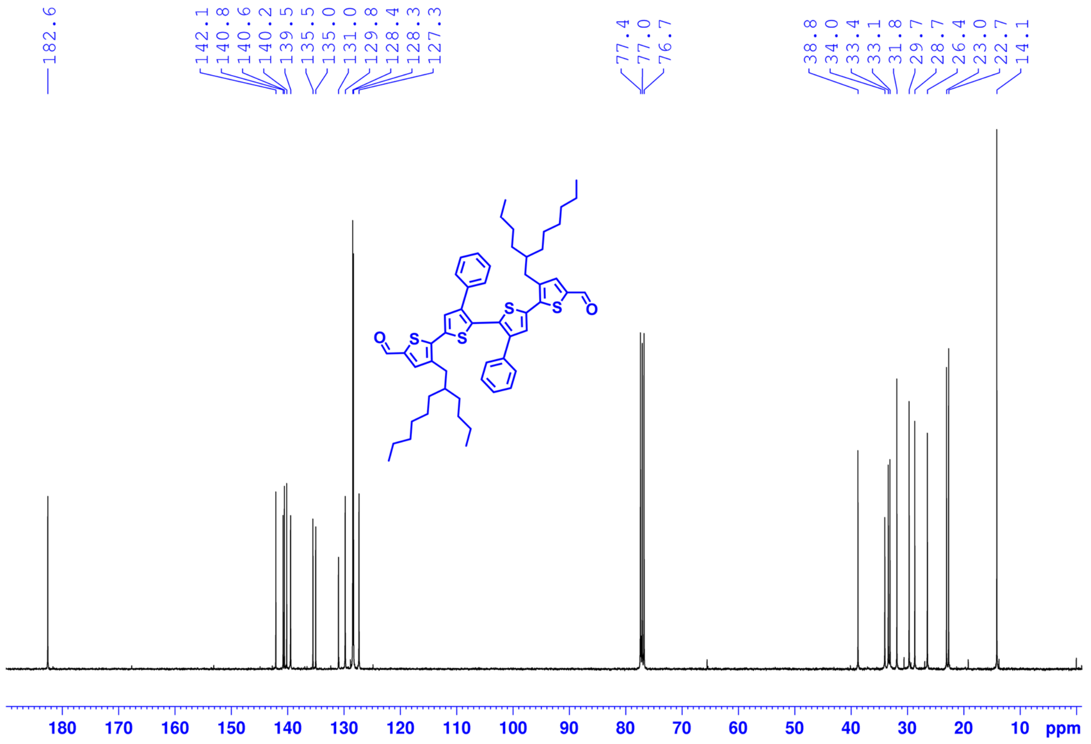
182.6
142.1 140.8 140.6 140.2 139.5 135.5 135.0 131.0 129.8 128.4 128.3 127.3
77.4 77.0 76.7
38.8 34.0 33.4 33.1 31.8 29.7 28.7 26.4 23.0 22.7 14.1
ppm

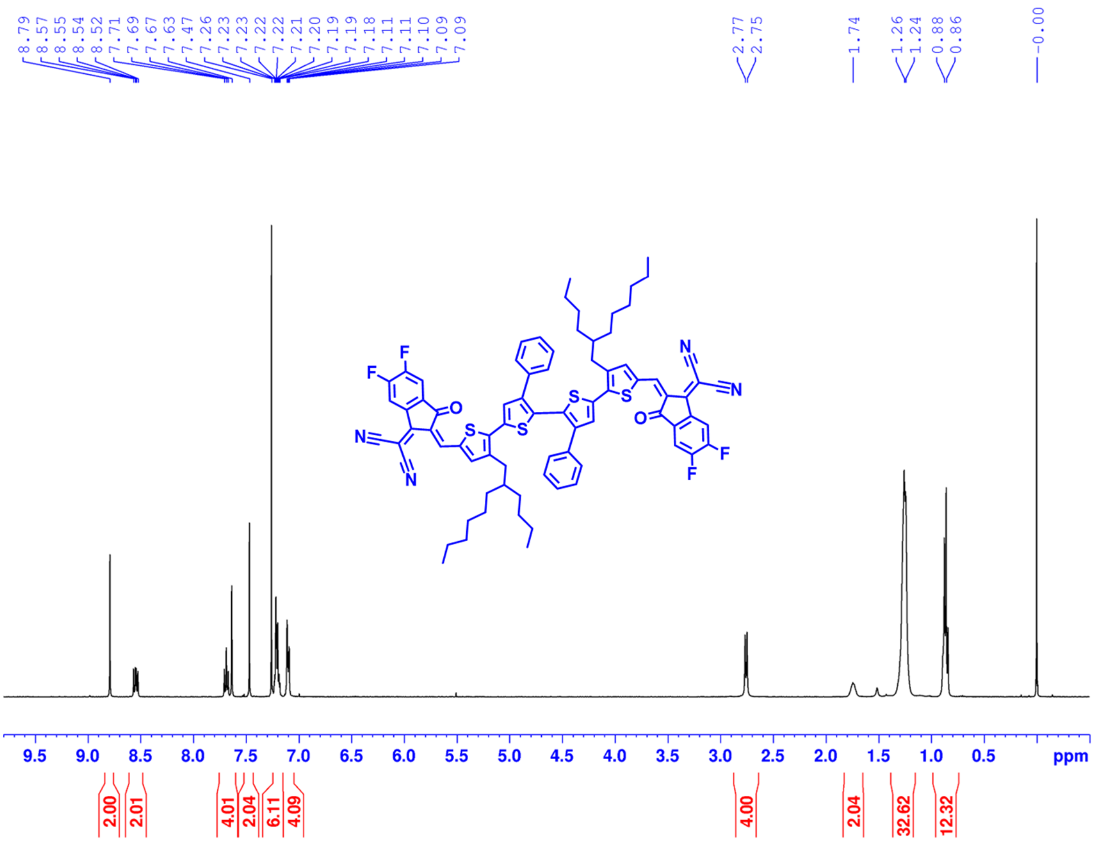

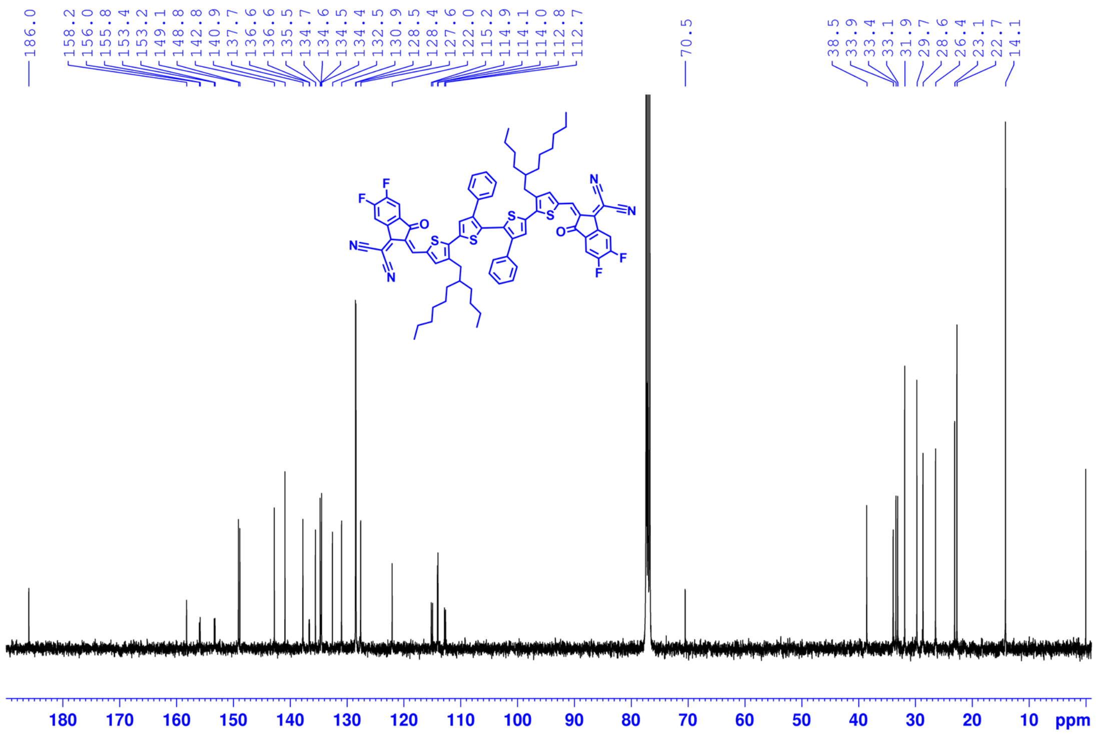

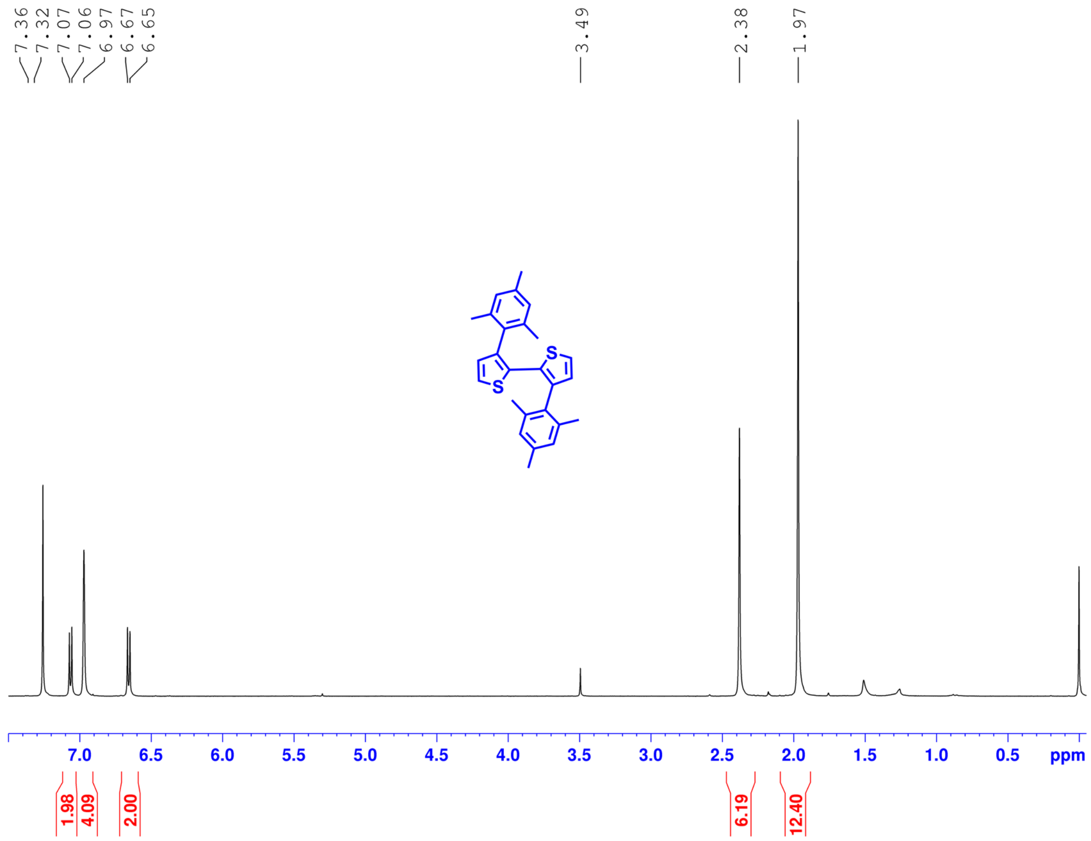
7.36
7.32
7.07
7.06
6.97
6.67
6.65
3.49
2.38
1.97
S
S
7.0
6.5
6.0
5.5
5.0
4.5
4.0
3.5
3.0
2.5
2.0
1.5
1.0
0.5
ppm
1.98
4.09
2.00
6.19
12.40

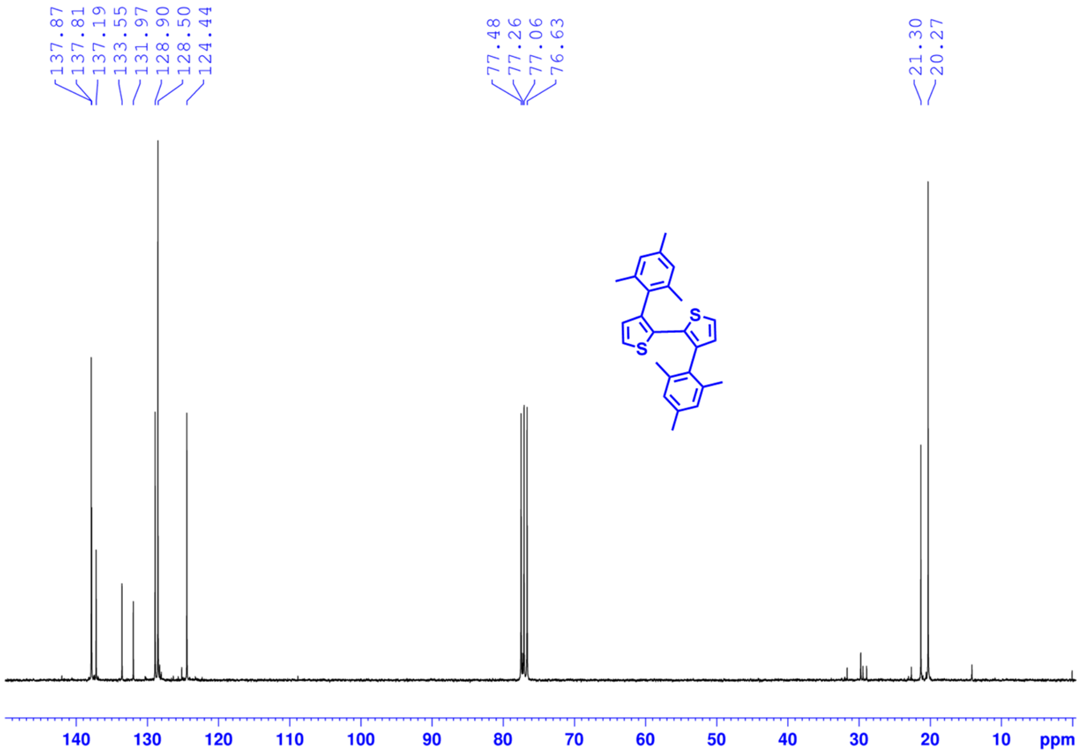
137.87
137.81
137.19
133.55
131.97
128.90
128.50
124.44
77.48
77.26
77.06
76.63
21.30
20.27
S
S
140
130
120
110
100
90
80
70
60
50
40
30
20
10
ppm

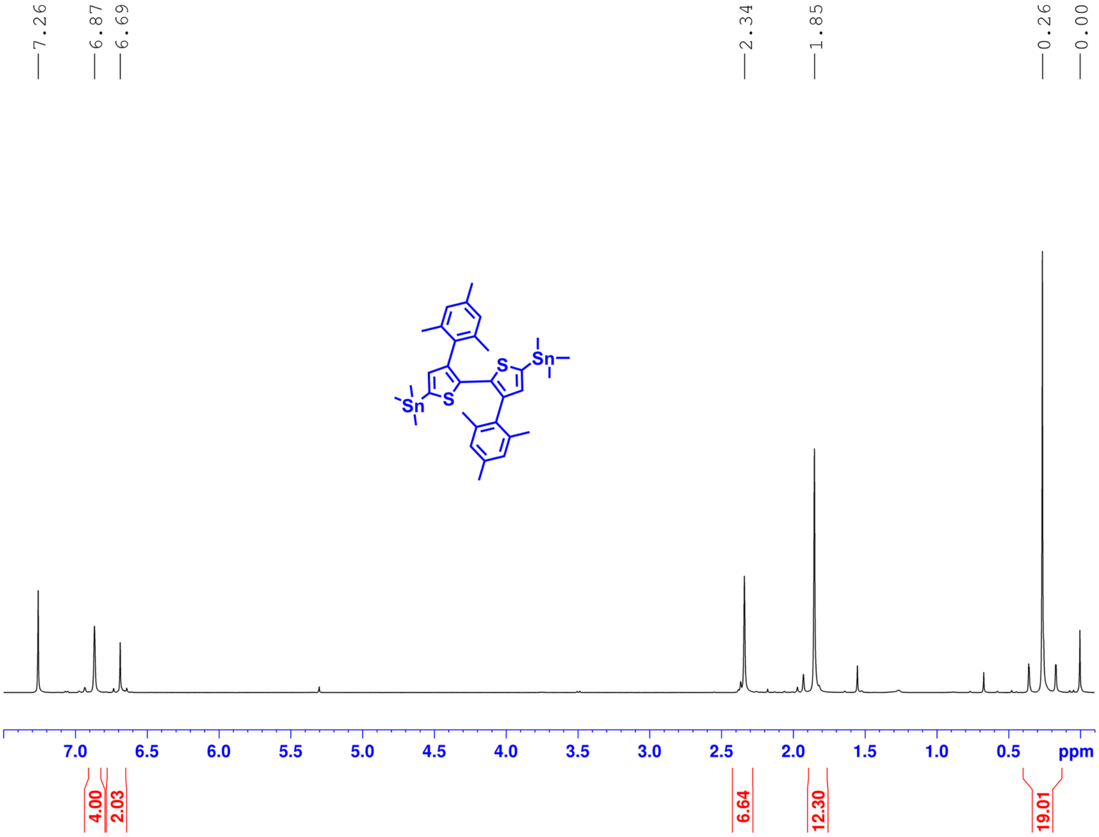
7.26
6.87
6.69
2.34
1.85
0.26
0.00
Sn
S
S
Sn
7.0
6.5
6.0
5.5
5.0
4.5
4.0
3.5
3.0
2.5
2.0
1.5
1.0
0.5
ppm
4.00
2.03
6.64
12.30
19.01

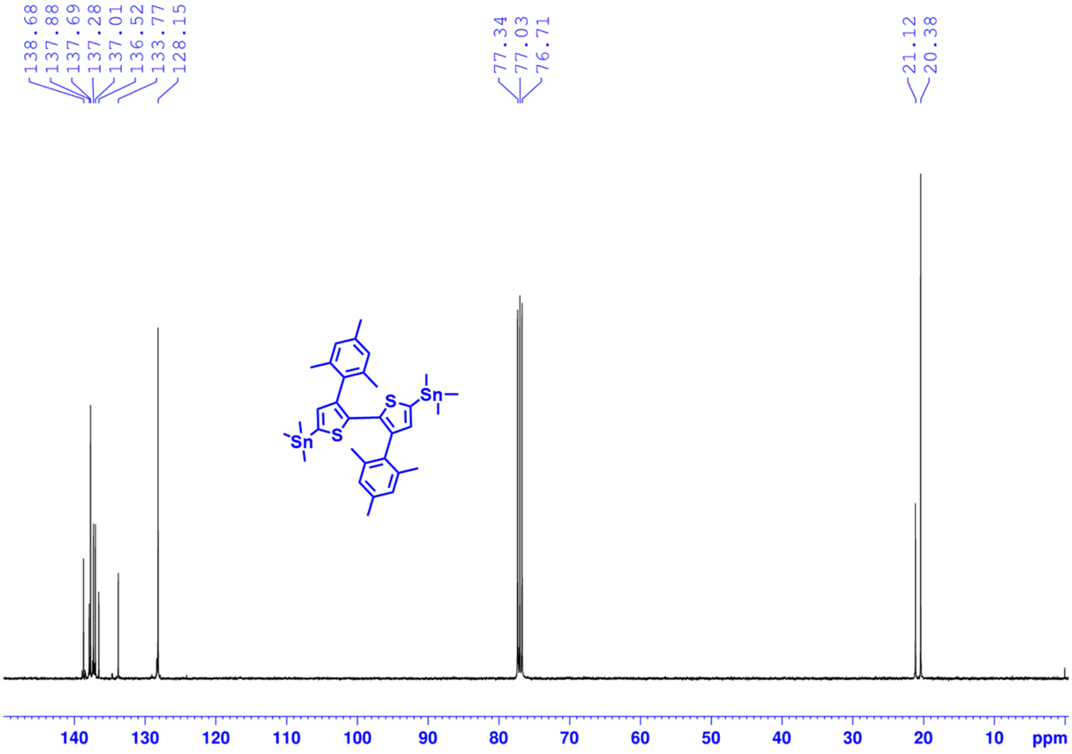
138.68
137.88
137.69
137.28
137.01
136.52
133.77
128.15
77.34
77.03
76.71
21.12
20.38
Sn
S
S
Sn
140
130
120
110
100
90
80
70
60
50
40
30
20
10
ppm

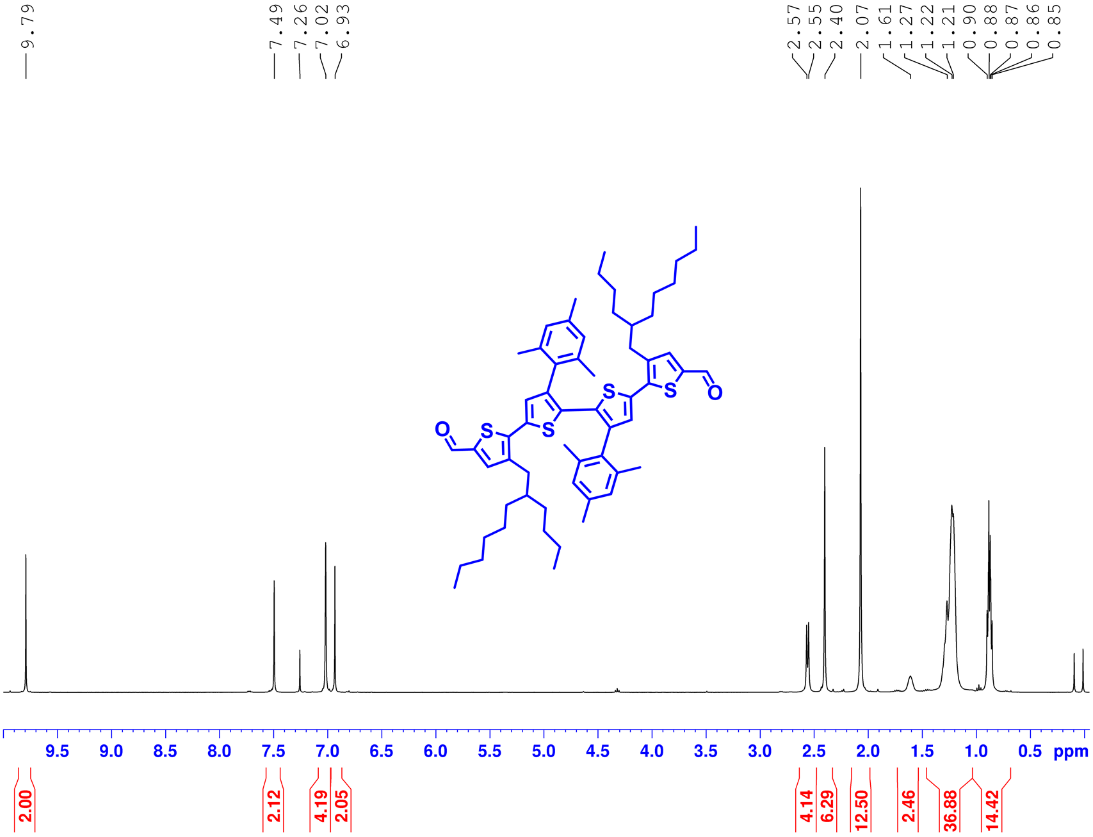
9.79
7.49
7.26
7.02
6.93
2.57
2.55
2.40
2.07
1.61
1.27
1.22
1.21
0.90
0.88
0.87
0.86
0.85
S
O
9.5 9.0 8.5 8.0 7.5 7.0 6.5 6.0 5.5 5.0 4.5 4.0 3.5 3.0 2.5 2.0 1.5 1.0 0.5 ppm
2.00
2.12
4.19
2.05
4.14
6.29
12.50
2.46
36.88
14.42

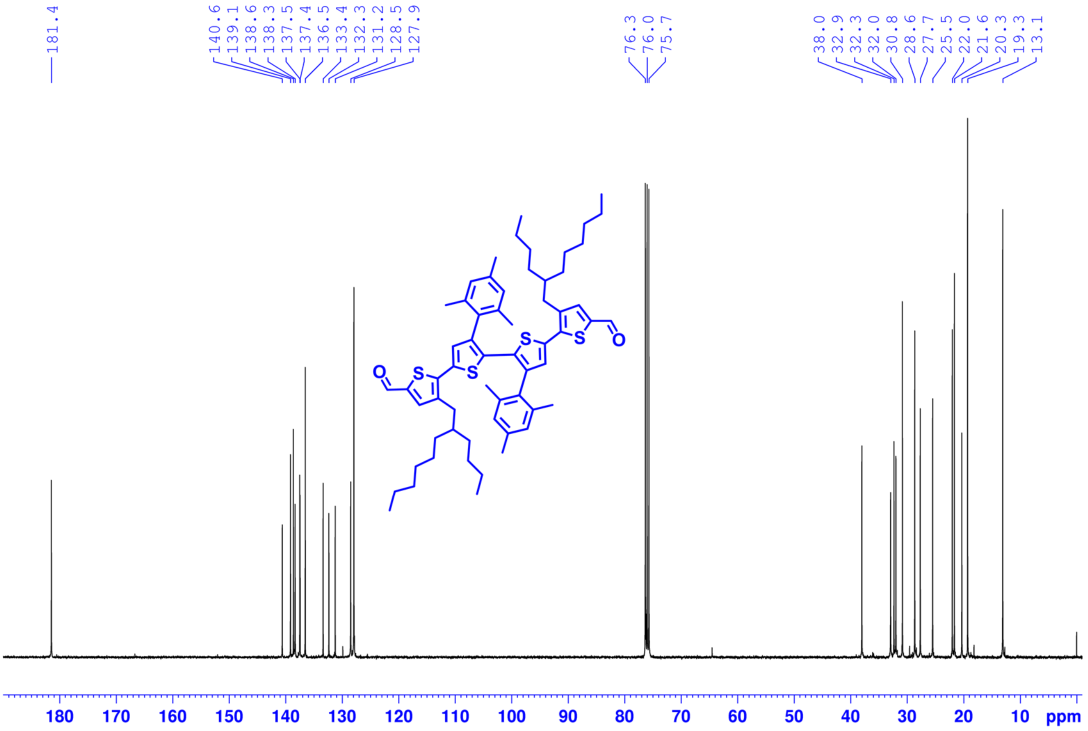
181.4
140.6
139.1
138.6
138.3
137.5
137.4
136.5
133.4
132.3
131.2
128.5
127.9
76.3
76.0
75.7
38.0
32.9
32.3
32.0
30.8
28.6
27.7
25.5
22.0
21.6
20.3
19.3
13.1
S
O
180 170 160 150 140 130 120 110 100 90 80 70 60 50 40 30 20 10 ppm

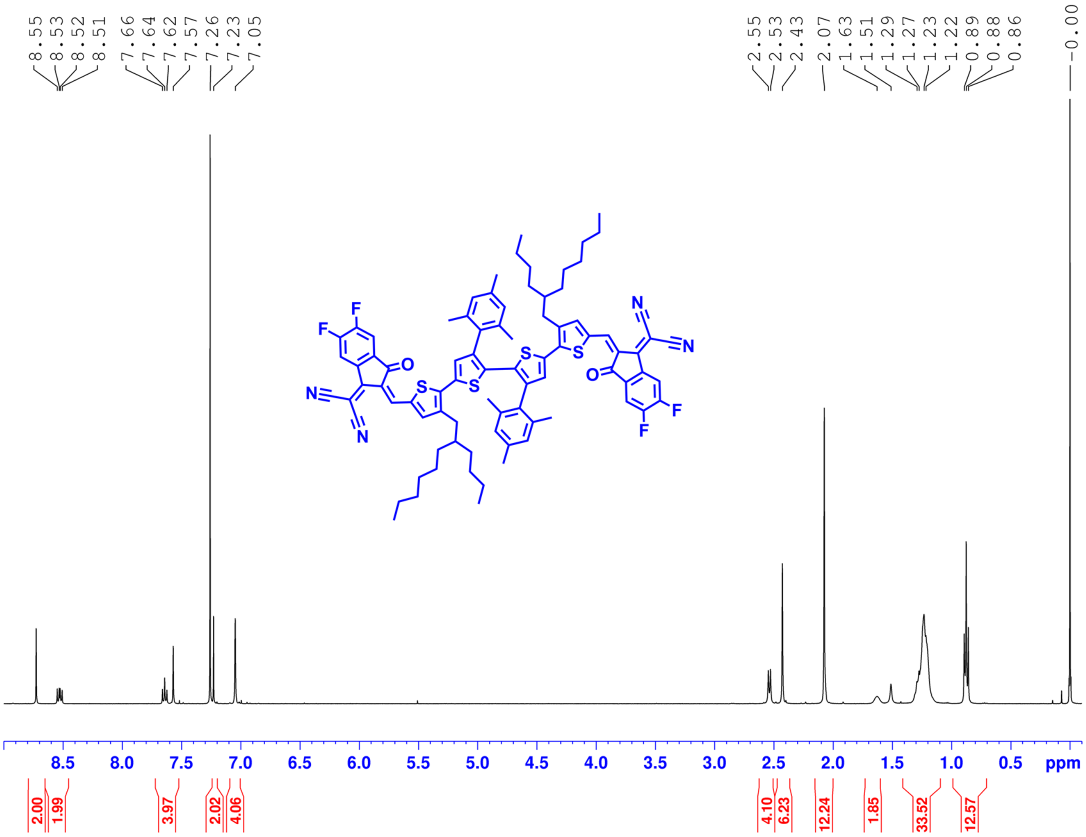
8.55
8.53
8.52
8.51
7.66
7.64
7.62
7.57
7.26
7.23
7.05
2.55
2.53
2.43
2.07
1.63
1.51
1.29
1.27
1.23
1.22
0.89
0.88
0.86
-0.00
F
F
O
S
S
S
S
N
N
N
N
O
F
F
8.5
8.0
7.5
7.0
6.5
6.0
5.5
5.0
4.5
4.0
3.5
3.0
2.5
2.0
1.5
1.0
0.5
ppm
2.00
1.99
3.97
2.02
4.06
4.10
6.23
12.24
1.85
33.52
12.57

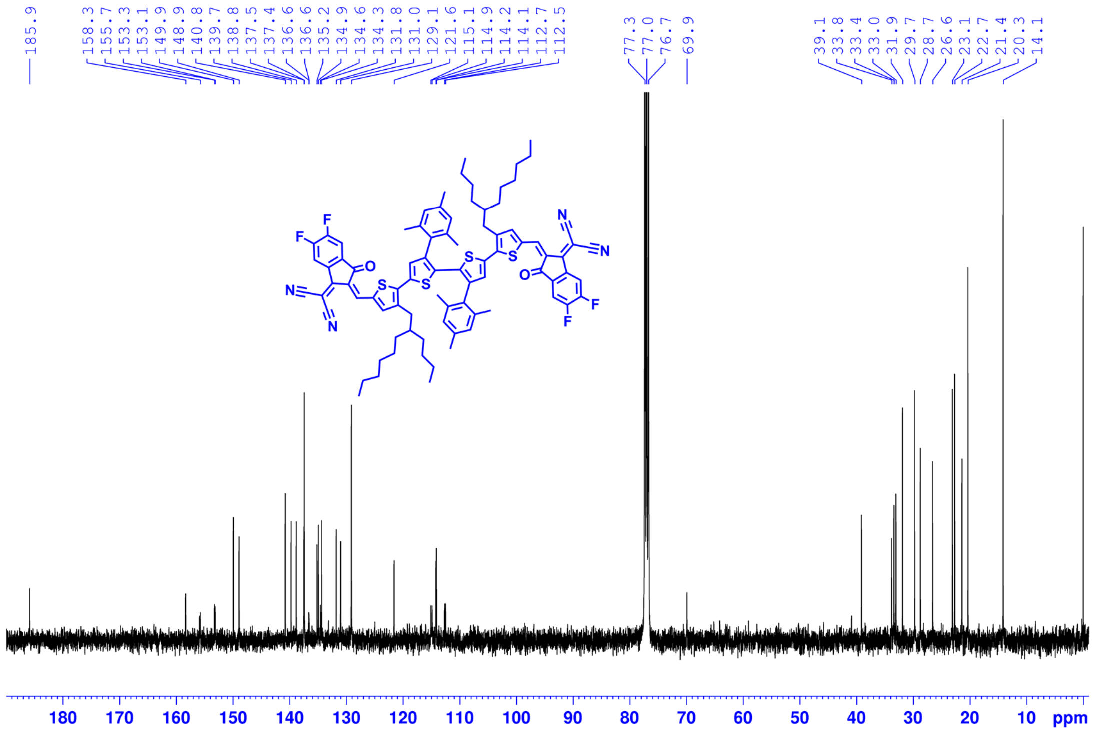
185.9
158.3
155.7
153.3
153.1
149.9
148.9
140.8
139.7
138.8
137.5
137.4
136.6
136.6
135.2
134.9
134.6
134.3
131.8
131.0
129.1
121.6
115.1
114.9
114.2
114.1
112.7
112.5
77.3
77.0
76.7
69.9
39.1
33.8
33.4
33.0
31.9
29.7
28.7
26.6
23.1
22.7
21.4
20.3
14.1
180
170
160
150
140
130
120
110
100
90
80
70
60
50
40
30
20
10
ppm

**SUPPLEMENTARY REFERENCES**

1. Schmidt A., Rahimi A. A versatile catalyst system for suzuki–miyaura syntheses of sterically hindered biaryls employing a cyclobutene-1,2-bis(imidazolium) salt. *Chem. Commun.* **46**, 2995 (2010).
2. Organ M. G., Calimsiz S., Sayah M., Hoi K. H., Lough A. J. Pd-peppsi-ipent: An active, sterically demanding cross-coupling catalyst and its application in the synthesis of tetra-ortho-substituted biaryls. *Angew. Chem., Int. Ed.* **48**, 2383-2387 (2009).
3. Wu Y., Dou Z., Zhang Z., Wang X., Yue G., Wu C., Chen H., Yin Z., Song X., He C., Yue G. Application of mesitylboronic acid and its esters in coupling reactions. *Chin. J. Org. Chem.* **38**, 2896-2926 (2018).
4. Frisch M. J., Trucks G. W., Schlegel H. B., Scuseria G. E., Robb M. A., Cheeseman J. R., Scalmani G., Barone V., Mennucci B., Petersson G. A., Nakatsuji H., Caricato M., Li X., Hratchian H. P., Izmaylov A. F., Bloino J., Zheng G., Sonnenberg J. L., Hada M., Ehara M., Toyota K., Fukuda R., Hasegawa J., Ishida M., Nakajima T., Honda Y., Kitao O., Nakai H., Vreven T., Montgomery Jr. J. A., Peralta J. E., Ogliaro F., Bearpark M. J., Heyd J., Brothers E. N., Kudin K. N., Staroverov V. N., Kobayashi R., Normand J., Raghavachari K., Rendell A. P., Burant J. C., Iyengar S. S., Tomasi J., Cossi M., Rega N., Millam N. J., Klene M., Knox J. E., Cross J. B., Bakken V., Adamo C., Jaramillo J., Gomperts R., Stratmann R. E., Yazyev O., Austin A. J., Cammi R., Pomelli C., Ochterski J. W., Martin R. L., Morokuma K., Zakrzewski V. G., Voth G. A., Salvador P., Dannenberg J. J., Dapprich S., Daniels A. D., Farkas Ö., Foresman J. B., Ortiz J. V., Cioslowski J., Fox D. J., Gaussian, Inc., Wallingford, CT, USA, **2009**.
5. Lu T., Chen F. Multiwfn: A multifunctional wavefunction analyzer. *J. Comput. Chem.* **33**, 580-592 (2012).
