# Supplementary material for: Completely non-fused electron acceptor with 3D-interpenetrated crystalline structure enables efficient and stable organic solar cell
Source: Nat Commun. 2021 Aug 24;12:5093. doi: 10.1038/s41467-021-25394-w (PMC8384863; doi:10.1038/s41467-021-25394-w)
Supplement: Supplementary file 2 — Solar Cells Reporting Summary [file 41467_2021_25394_MOESM2_ESM.pdf]

nature research

Corresponding author(s): Jianhui Hou

Solar Cells Reporting Summary

Nature Research wishes to improve the reproducibility of the work that we publish. This form is intended for publication with all accepted papers reporting the characterization of photovoltaic devices and provides structure for consistency and transparency in reporting. Some list items might not apply to an individual manuscript, but all fields must be completed for clarity.

For further information on Nature Research policies, including our data availability policy, see Authors & Referees.

Experimental design

Please check: are the following details reported in the manuscript?

1. Dimensions

Item	Yes	No	Comment
Area of the tested solar cells	☒	☐	Area of the tested solar cells is provided in methods, section"Device characterization and measurement".
Method used to determine the device area	☒	☐	The method is provided in methods, section"Device characterization and measurement".

2. Current-voltage characterization

Item	Yes	No	Comment
Current density-voltage (J-V) plots in both forward and backward direction	☐	☒	Just J-V plot in forward direction since there is no hysteresis in organic solar cells.
Voltage scan conditions<br>*For instance: scan direction, speed, dwell times*	☒	☐	The scan conditions are provided in methods, section"Device characterization and measurement".
Test environment<br>*For instance: characterization temperature, in air or in glove box*	☒	☐	Test environment is provided in methods, section"Device characterization and measurement".
Protocol for preconditioning of the device before its characterization	☐	☒	No preconditioning protocol.
Stability of the J-V characteristic<br>*Verified with time evolution of the maximum power point or with the photocurrent at maximum power point; see ref. 7 for details.*	☐	☒	there is no corresponding testing equipment

3. Hysteresis or any other unusual behaviour

Item	Yes	No	Comment
Description of the unusual behaviour observed during the characterization	☐	☒	No. In general, organic solar cells do not have hystereisis problems.
Related experimental data	☐	☒	No.

4. Efficiency

Item	Yes	No	Comment
External quantum efficiency (EQE) or incident photons to current efficiency (IPCE)	☒	☐	EQE curves are shown in Figure 5b.
A comparison between the integrated response under the standard reference spectrum and the response measure under the simulator	☐	☒	No
For tandem solar cells, the bias illumination and bias voltage used for each subcell	☐	☒	Our cells were only fabricated for single-junction solar cells.

5. Calibration

Item	Yes	No	Comment
Light source and reference cell or sensor used for the characterization	☒	☐	Relative information is provided in methods, section"device characterization and measurement".
Confirmation that the reference cell was calibrated and certified	☒	☐	Relative information is provided in methods, section"device characterization and measurement".

Calculation of spectral mismatch between the reference cell and the devices under test

☐ Yes
☒ No

The spectral mismatch factor was determined at National Institute of Metrology, China (NIM). We do not have the detailed information for the method.

6. Mask/aperture

Size of the mask/aperture used during testing

☒ Yes
☐ No

Size of the mask is provided in methods, section"device characterization and measurement".

Variation of the measured short-circuit current density with the mask/aperture area

☒ Yes
☐ No

Information is provided in table 1.

7. Performance certification

Identity of the independent certification laboratory that confirmed the photovoltaic performance

☒ Yes
☐ No

Certified results are provided in the Supplementary Figure7.

A copy of any certificate(s)
Provide in Supplementary Information

☒ Yes
☐ No

Certified results are provided in the Supplementary Figure7.

8. Statistics

Number of solar cells tested

☒ Yes
☐ No

Number of cells tested is provided in Table 1

Statistical analysis of the device performance

☒ Yes
☐ No

Statistical results of the devices are listed in Table 1

9. Long-term stability analysis

Type of analysis, bias conditions and environmental conditions
For instance: illumination type, temperature, atmosphere humidity, encapsulation method, preconditioning temperature

☒ Yes
☐ No

The bias conditions for Long-term stability analysis are provided in Figure 7d and the corresponding main text.
